# Supplementary material for: An instructive role for Interleukin-7 receptor α in the development of human B-cell precursor leukemia
Source: Nat Commun. 2022 Feb 3;13:659. doi: 10.1038/s41467-022-28218-7 (PMC8814001; doi:10.1038/s41467-022-28218-7)
Supplement: Supplementary file 1 — Supplementary Information [file 41467_2022_28218_MOESM1_ESM.pdf]

An instructive role for Interleukin-7 receptor α in the development of human B-cell precursor leukemia

Supplementary information

Supplementary tables

			BB		CRLF-GFP		CRLF-IL7RAwt		CRLF-IL7RAins		IL7RAwt		IL7RAins		unt
exp	cage	CB Batch	engrafted	Not engrftted	engrafted	Not engrftted	engrafted	Not engrftted	engrafted	Not engrftted	engrafted	Not engrftted	engrafted	Not engrftted	Engrafted
1	9	I		1				1		1					1
1	10	II		1		1	1		1						
1	11	II		1	1			1	1						1
1	12	IV		1				1		1					1
1	13	V	1		1			1							1
1	14	V	1		1										1
1	15	VI		1		1									
1	17	VII	1			1	1			1					1
1	18	VII	1			1	1		1						1
2	1	VIII	1					1	1						1
2	2	IX (P)	1				1		1						
2	3	X	1		1										1
2	4	X	1				1		1						
2	5	XI		1	1		1		1						1
2	6	XII (P)	1				1		1						
2	7	XIII (P)	1			1			1						
2	8	XIV	1		1				1						
2	9	XIV		1					1						
3	1	XV	1				1			1	1		2		1
3	2	XVI (P)	1				1		1		1		1		
3	3	XVI			1						1		1		1
3	4	XVI (P)	1		1		1		1	1			1 (L)		
3	5	XVII	1				1		1		1		1		
3	6	XVII			1						1		1		1
3	7	XVIII		1										1	
3	8	XIX		1				1	1			1	1		
3	9	XX		1							1		1		
3	10	XX	1						1			1	1		1
3	11	XX	1							1	1		1		1
3	12	XXI	2								2		1	1	
3	13	XXII	1								1		1		
3	14	XXII	1								1		1		
		SUM	31		14		17		22		13		16		15

Supplementary table 1: Transplantation table – numbers indicate numbers of mice transplanted in each condition. Batches marked with (P) developed $CD10^{+}CD34^{+}$ pre-leukemic population. (L) indicates original pre-leukemic mouse from which spontaneous leukemia developed. Engraftment rules: More than 2% hCD45 and more than 3% distinct transduced $CD19^{+}$ population. BB=backbone vector; unt= untransduced

Genomic Position	Tumor Call	Effect
chr1:6128316-6550966	./.:1:0	DEL
chr2:88855243-88914861	./.:0:0	DEL
chr2:88916530-89021436	./.:1:0	DEL
chr2:89022486-89032415	./.:0:0	DEL
chr2:89034492-89055526	./.:1:0	DEL
chr2:89056678-89082665	./.:0:0	DEL
chr2:89085229-89088777	./.:1:0	DEL
chr2:89089906-89108033	./.:2:0	DEL
chr2:89109152-89244680	./.:1:0	DEL
chr2:89246139-89250404	./.:2:0	DEL
chr2:89251664-89298147	./.:1:0	DEL
chr2:89940432-90095478	./.:1:0	DEL
chr3:138798511-138808252	./.:1:0	DEL
chr3:82584435-82600604	./.:3:1	DUP
chr7:38256528-38329654	./.:0:0	DEL
chr7:38331081-38361383	./.:1:0	DEL
chr9:137532898-137572364	./.:1:0	DEL
chr9:5633386-37485713	./.:1:0	DEL
chr14:105866868-106032920	./.:3:0	DUP
chr14:106034543-106153593	./.:2:0	DEL
chr14:106155125-106405332	./.:3:1	DUP
chr14:106514372-106554068	./.:3:1	DUP

Within 88825371-90316061
IGK locus

Within 105536746-106879844
IGH locus

Supplementary table 2: Leukemia genomic structural variations. Leu*kemic* cells and batch matched backbone transduced engrafted cord blood (representing germline) were sequenced (whole genome sequencing 60x). The table depicts major structural variations between the samples. DEL-deletion, DUP-duplication.

		IL7RAins		BB		Untransduced
EXP	CB Batch	sgCDKN2A	sgLuciferase	sgCDKN2A	sgLuciferase	
Exp4	25-7-G	4	2	1	1	1
Exp4	25-7-B	2 (1L)	1	1	1	1
Exp4	25-7-Y	2	1	1	1	1
Exp 5	26-7-1	2 (1L)	1	1	1	
Exp 5	26-7-2	2 (1L)		1	1	1
Exp 6	1-8-M	1	1	1	1	1
Exp 6	1-8-193	1	1	1		1

Supplementary table 3**:** transplantation table of the CDKN2A CRISPR editing experiments. Numbers indicate numbers of mice transplanted in each condition. (L) indicates leukemia development. CB- cord blood.

sample	Primer set	Clonotype
O1	IGH-DJ	DJ:Dh-Jh D2-21 -6/4/-2 J6 VSILWW*LH#YYYYYGMDVW
	IGH-DJ	DJ:Dh-Jh D2-21 -5/0/-1 J5 VSILWW*LLNWFDPW
	intron-Kde	intron-Kde intron -3/1/-15 Kde PCVCPINAAVASFP##PGR
L5	IGH-DJ	DJ:Dh-Jh D6-25 -0/23/-3 J5 VGYSSGYHPRYRER##WFDPW
	IGH-VJ-FR1	VJ:Vh-(Dh)-Jh V3-43 -3/9/-1 J6 CAKGRT#YYYYYYMDVW
	IGK-VJ-Kde	Vk-Kde V4-1 -3/2/-5 Kde CQQYYSTP#PSGSPGR
PA4	IGH-VJ-FR1	VJ:Vh-(Dh)-Jh V3-13 -0/20/-5 J6 CARDSREAGT#YYYYGMDVW
	IGK-VJ-Kde	VJ:Vk-Jk V2D-26 -6/3/-13 J4 CMQDAQE
	TRD	VD:Vd-Dd3 V2 -21/2/-2 D3 HGGYY

Supplementary table 4: Clonal V(D)J markers of Leukemic cells. Markers of clonal populations sorted (CD45+/CD34+) from Leukemic mice.

antigen	Clone	Manufacturer	fluorophore	Dilution	Cat number
CD45	5B1	Miltenyi	Vio Green	1:50	130-096-906
	HI30	Biolegend	Pacific Blue	1:50	304029
	HI30	Biotest	APC	1:50	17-0459-42
CD127(IL7RA)	R34.34	Beckman Coulter	APC-Alexa 700	2:50	A71116
	eBioRDR5	eBioscience	Super Bright 780	2:50	78-1278-42
	A019D5	Biolegend	BV421	2:50	351310
CD19	J3-119	Beckman Coulter	ECD	1:50	A07770
	J3-119	Beckman Coulter	APC-Alexa750	1:50	A78838
IgM	CH2	exbio	APC	1:100	1A-320-C100
CD10	ALB1	Beckman Coulter	PC7	1:50	A465527
CD34	BIRMA-K3	DAKO/Agilent	RPE	1:100	C723850-2
	8G12/HPCA2	BD Pharmingen	APC	1:100	345804
CD16/32	93	Biolegend	none	1:50	101326
TSLPR	ID3	Biolegend	PE	1.5:50	322906
	IB4	Biolegend	PE	1.5:50	322806
Nucleic acid	N/A	BD Pharmingen	7AAD	1:50	559925
LIVE/DEAD fixable Dead cells staining	N/A	Molecular probes	Near IR/Violet	1:1000	L34975

Supplementary table 5: Antibodies and markers for flow cytometry. All antibodies directed against human antigens

Protein	Clone	Manufacturer	Metal Isotope	Staining	Diluition (in 100 µL)
4EBP1(pT36/T46)	236B4	Cell Signaling Technology	Nd144	Intracellular	1 µL
Akt (pS473)	D9E	Cell Signaling Technology	Tb159	Intracellular	1 µL
BTK (pY551/511)	24A/BTK	BD Biosciences	Yb174	Intracellular	2 µL
cCaspase3	C92-605	BD Biosciences	Ho165	Intracellular	1 µL
CD10	HI10a	Biolegend	Gd156	Surface	1 µL
CD127	A019D5	Biolegend	Dy162	Surface	1.5 µL
CD16	3G8	Fluidigm	Bi209	Surface	2 µL
CD179a	HSL96	Biolegend	Sm149	Intracellular	0.3 µL
CD179b	HSL11	Biolegend	Gd158	Intracellular	0.5 µL
CD19	H1B19	Biolegend	Nd142	Surface	0.5 µL
CD20	2H7	Biolegend	Sm147	Surface	0.5 µL
CD22	HIB22	Biolegend	Nd143	Surface	1 µL
CD235	HIR2	Biolegend	In115	Surface	0.25 µL
CD24	ML5	Biolegend	Gd160	Surface	1 µL
CD3	UCHT1	Biolegend	Er170	Surface	0.25 µL
CD34	581	Biolegend	Nd148	Surface	0.25 µL
CD38	HIT2	Biolegend	Er168	Surface	0.5 µL
CD43	CD43-10G7	Biolegend	Er167	Surface	0.25 µL
CD45 human	HI30	Fluidigm	Y89	Surface	0.25 µL
CD45 mouse	30F11	Biolegend	In113	Surface	0.125 µL
CD79b	CB3-1	Biolegend	Nd146	Surface	0.5 µL
cPARP	F21-852	BD Biosciences	La139	Intracellular	0.25 µL
Creb (pS133)	87G3	Cell Signaling Technology	Yb176	Intracellular	5 µL
CRLF2	1A6	eBioscience	Dy161	Surface	1 µL
CyclinA (total)	BF-683	BD Biosciences	Sm154	Intracellular	0.25 µL
CyclinB1 (total)	GNS-1	BD Biosciences	Dy164	Intracellular	1 µL
Erk1/2 (pT202/pY204)	D13-14-4E	Cell Signaling Technology	Yb173	Intracellular	1 µL
Glucocorticoid Receptor	D8H2	Cell Signaling Technology	Eu151	Intracellular	2 µL
GFP	SF12.4	Fluidigm	Tm169	Intracellular	1 µL
HistoneH3 (pS28)	HTA28	Biolegend	Ce140	Intracellular	0.25 µL
IgHintracellular	polyclonal	Novus	Eu153	Intracellular	0.5 µL
IgH surface	MHM-98	Fluidigm	Yb172	Surface	0.25 µL
Ikaros (total)	D10E5	Cell Signaling Technology	Nd145	Intracellular	2 µL
Ki67	B56	BD Biosciences	Sm152	Intracellular	1 µL
PU.1	9G7	Cell Signaling Technology	Gd157	Intracellular	1 µL
RB (pS807/811)	J112-906	BD Biosciences	Er166	Intracellular	0.25 µL
rpS6 (pS235/pS236)	N7-548	BD Biosciences	Lu175	Intracellular	1 µL
SRC (pY418)	K98-37	BD Biosciences	Pr141	Intracellular	2 µL
STAT5 (pY694)	47	BD Biosciences	Gd155	Intracellular	0.5 µL
Syk (pY319/pY352)	17a	BD Biosciences	Yb171	Intracellular	1 µL
TdT	E17-1519	BD Biosciences	Dy163	Intracellular	1 µL

Supplementary table 6: Antibodies and reagents for mass cytometry.

Supplementary figures

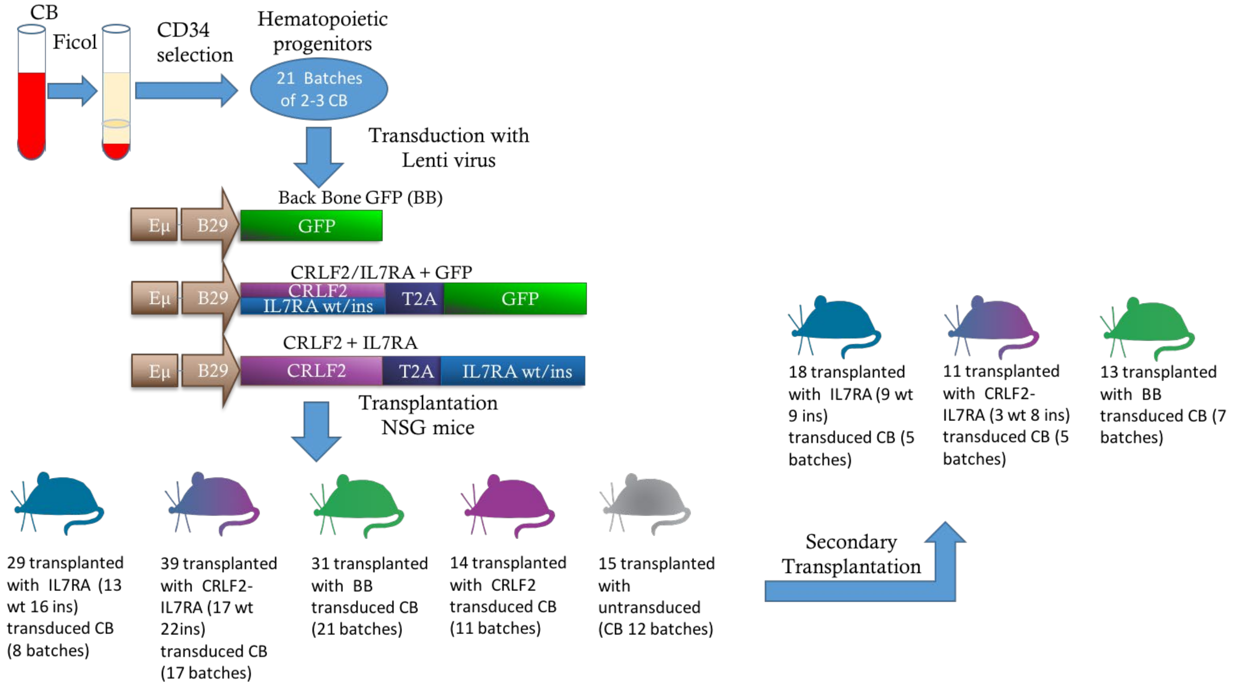

Supplementary figure 1: Experimental flow: Cord Blood (CB) samples were ficolled and subjected to CD34 selection. Cells were then transduced with lentivectors directing expression of GFP and bi-cistronic expression of combinations of CRLF2, IL7RAwt, mutated IL7RA with inframe insertion encoding the amino acids PPCL. Under B-cell minimal promoter/enhancer (pRRL Eμ B29). Transduced cells from 2-3 CB donors were mixed and transplanted in sub-lethally irradiated 5-8 weeks old female NOD/LtSz-scid IL2Rγnull (NSG) mice. 24-32 weeks after transplantation mice are sacrificed and whole BM cells (with estimated 1-2X10^5 transduced CD45 cells calculated after staining) were re-transplanted into naïve irradiated NSG mice.

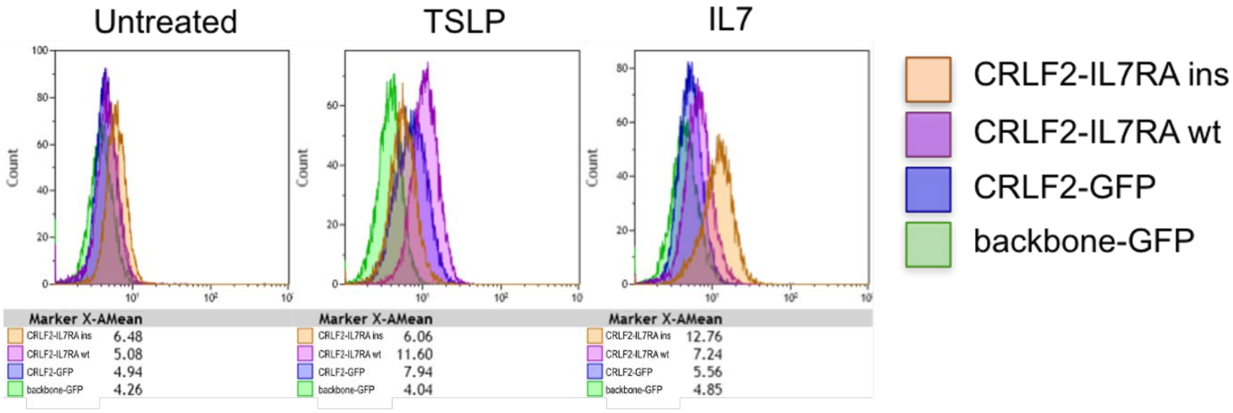

Supplementary figure 2: CRLF2 and IL7RA expression from lentivirus transduction facilitates cytokine response. Flow cytometer histogram of phospho-STAT5 in transduced 018Z (BCP-ALL) cells after activation with TSLP (2ng/ml) or IL7 (2ng/ml). Mean fluorescent intensities are portrayed in the histogram and values are listed below. The experiment was performed once in the above cell lines and verified twice in Ba-F3 cell line.

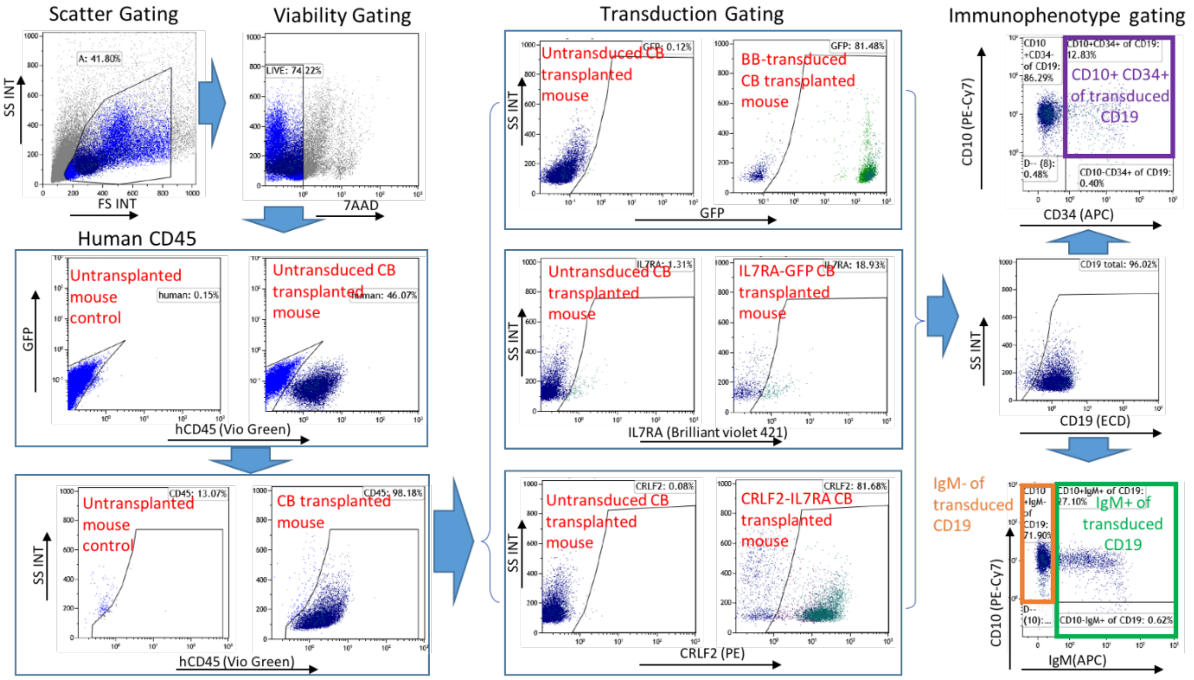

Supplementary figure 3: Gating strategy for immunophenotying. Stained single cell suspension from bone marrow and spleen was analyzed. Lymphocytes were gated in a Forward scatter (FS) vs. side scatter (SS) dot plot. Live cells (7AAD-) cells were then gated. To eliminate mouse tissue background in the violet laser, negative gate is set according to untransplanted mouse. Human cells are marked by hCD45 (Vio green/APC) gate which was set based on untransplanted mouse. Transduced cells were gated using CRLF2 (PE)/IL7RA (BV 421/super bright 780) antibodies or GFP expression. Negative gates were set according to untransduced transplanted mouse cells or according to fluorescent minus one (FMO) staining in the case of IL7RA transduction (as antibodies detect also endogenous expressed IL7RA). $CD10^+$(PC7)$CD19^+$(ECD/APC-Alexa750) or $CD19^+$ were set from transduced population. $CD34^+$(APC)$CD10^+$ (for figure 2a) and IgM^+ or $CD10^+$ IgM^- (for figure 1a,b and supplementary figure 4a,b) were gated from transduced $CD19^+$ cells (these values were collected from 2 different staining panels when IgM and CD34 were interchangeably used). Gates were set according to FMO staining.

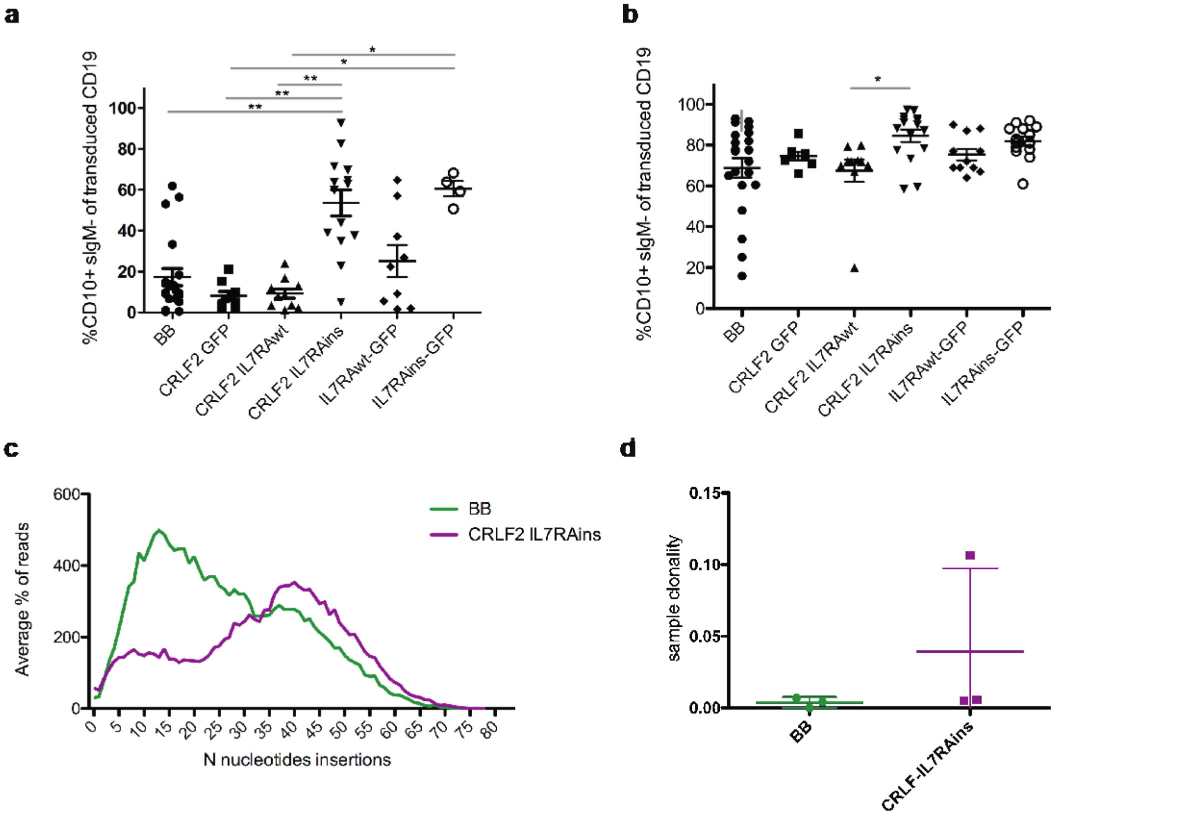

Supplementary figure 4: Early B-cell differentiation, Clonality and N- nucleotide insertions in activated IL7RA and control backbone transduced cells. (A,B) In vivo B-cell Differentiation of human CB CD34^{+} pro-pre-B-cells (CD10+ sIgM-) out of total B-cells (CD19+) from spleen (a) and BM (b) of engrafted mice expressing GFP (BB) [n=20 (a), n=21(b)] , CRLF2-GFP (n=9 (a), n=9 (b)] , CRLF2-IL7RAwt [n=10 (a), n=10 (b), CRLF2-IL7RAins [n=14 (a), n=16 (b)], IL7RAwt-GFP [n=9 (a), n=11(b)] and IL7RAins-GFP [n=4 (a) n=14 9 (b)] Dot plots show sample scatter with mean +/- SEM. Each dot represents analysis of single mouse. Statistical analyses were performed using Kruskal-Wallis non-parametric test (Gaussian Approximation) (a) $p<0.0001$ Kruskal-Wallis statistic = 28.7 (b) $p<0.0035$ Kruskal-Wallis statistic = 17.57 Gray linkers indicate statistically significant difference (* $p<0.05$, ** $p<0.01$, ***$p<0.001$) between groups in Dunn's post-hoc analysis significance level α=0.0 between groups with Dunn's post-hoc analysis significance level α=0.05. c) Histogram depicting average percent of reads for each N-nucleotides insertion quantity that was measured in each sample for each condition (Backbone /CRLF2-IL7RAins transduced transplanted CD19/CD10 sorted cells n=3 per condition). Statistical analysis was performed using two way repeated measurements ANOVA test Difference p values: Interaction $p< 0.0001$, N nucleotide insertion $p<0.0001$ transgene p=0.148, subjects (matching) $p<0.0001$. d) Dot plots *representing* total sample clonality of BM CD10^{+} and CD19^{+} sorted cells. Each dot represent a value of a single sample. Lines represent mean +/- SEM of BB (n=3) and CRLF2-IL7RAins (n=3). Statistical analyses were performed using two tailed t-test, the difference is not significant p=0.3. Source Data is provided in a Source Data file.

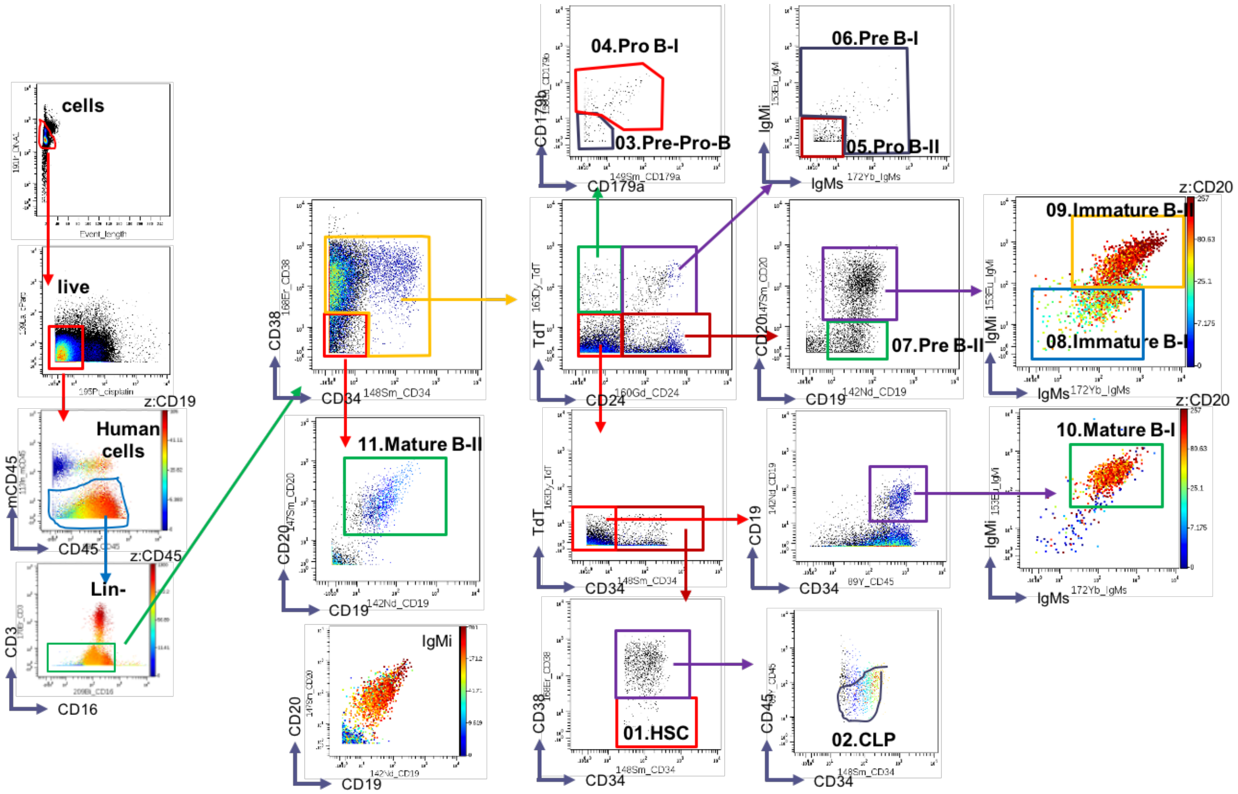

Supplementary figure 5: Gating strategy of mass cytometry analysis for B-cell developmental classifier. Healthy BMs were used to run the developmental classifier. The left four plots indicate the gating strategy used to obtain live lineage-negative cells (mCD45$^-$/CD3$^-$/CD16$^-$) that represent the starting population for the subsequent analysis of pre-leukemic and leukemic samples with the developmental classifier.

The 11 healthy developmental populations that were used as reference for the developmental classifier are accordingly numbered in the gating strategy. Arrows indicate hierarchical gating. Plots are colored by either cell density or marker expression as indicated by the z-axis on the right.

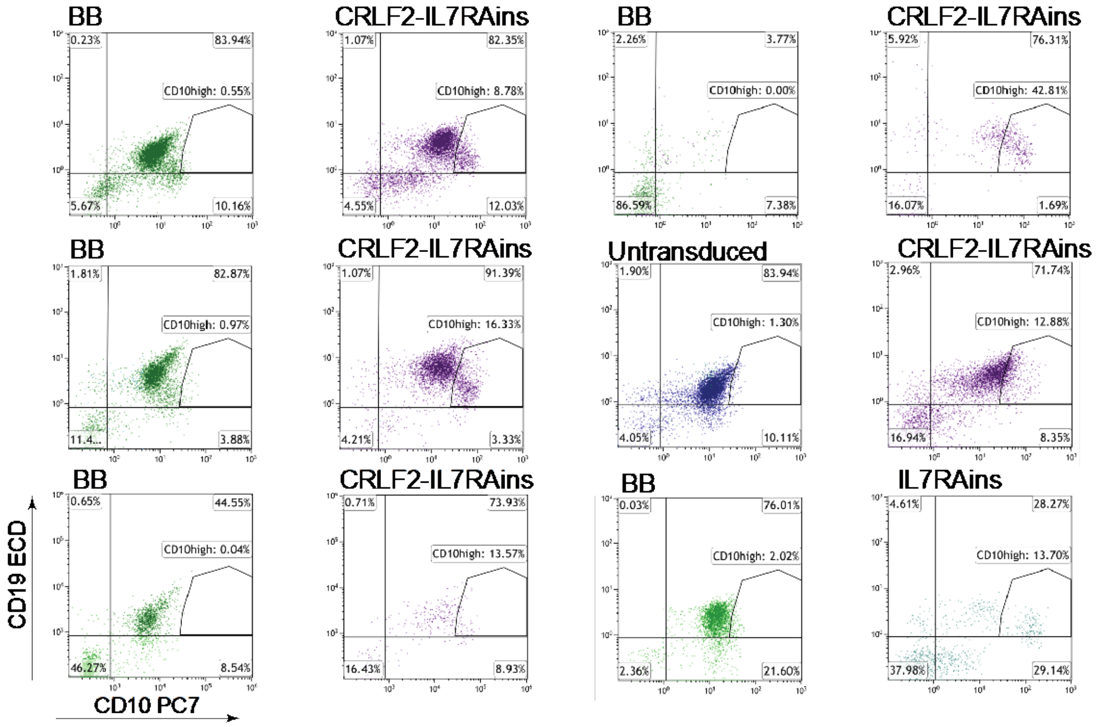

Supplementary figure 6: Unique CD10^{high}CD19^{+} population in IL7RAins transduced cells. Flow cytometry immunophenotyping plots of engrafted backbone and CRLF2-IL7RAins or IL7RAins transduced cells. Paired samples are cord blood batch matched. BB-backbone GFP control.

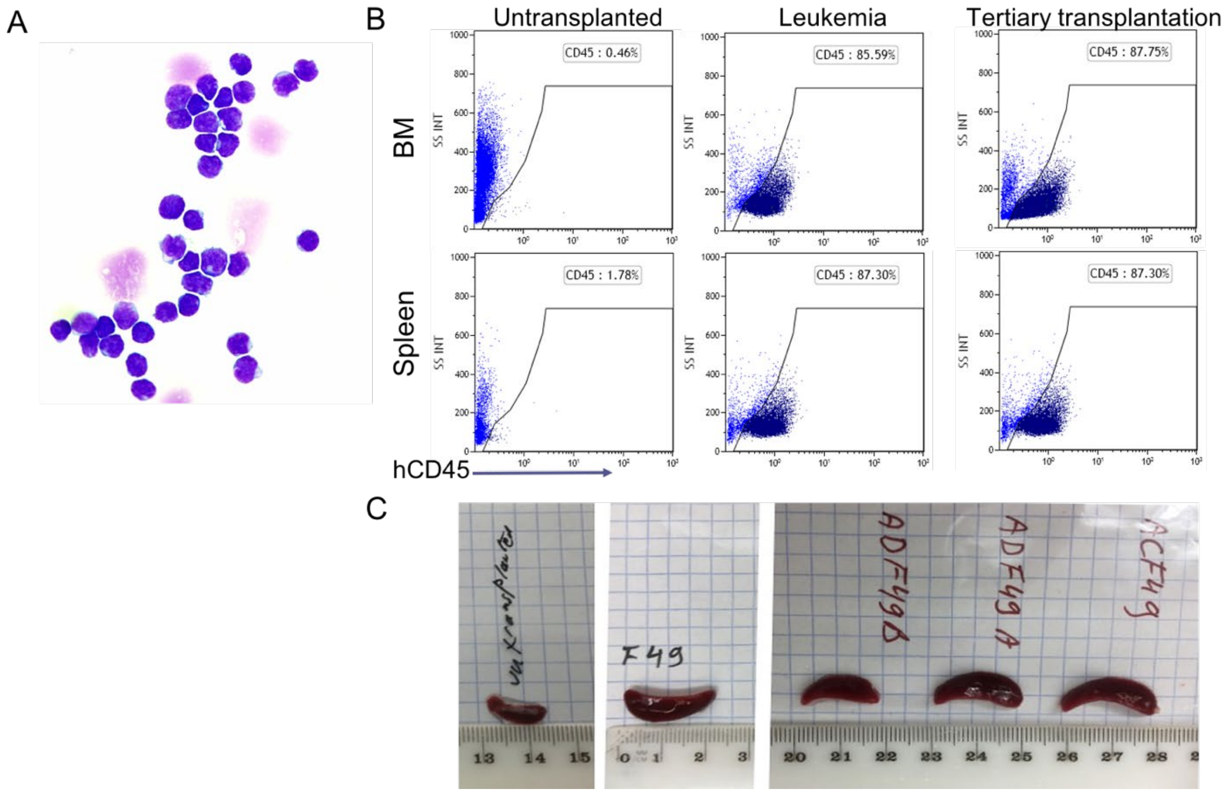

Supplementary figure 7: Human engraftment in leukemic mice. A) GIEMSA staining of cytospin from BM of the leukemic mouse. B) Flow cytometry charts of BM and spleen of untransplanted mouse (left) secondary IL7RA engrafted mouse that developed leukemia (center) and tertiary transplanted mouse that was engrafted with cells from spleen of leukemic mouse (right) C) Pictures depicting spleen size at sacrifice of untransplanted mouse (left) secondary IL7RA engrafted mouse that developed leukemia (center) and three tertiary transplanted mouse that were engrafted with cells from spleen of leukemic mouse (right).

V-(D)-J rearrangement summary for query sequence (multiple equivalent top matches, if present, are separated by a comma):

Top V gene match	Top D gene match	Top J gene match	Chain type	stop codon	V-J frame	Productive	Strand
IGHV3-15*01,IGHV3-15*02	IGHD3-10*01	IGHJ4*02	VH	Yes	Out-of-frame	No	+

V-(D)-J junction details based on top germline gene matches:

V region end	V-D junction*	D region	D-J junction*	J region start
ACAGA	TGGGGCGC	TACTATGGTTCGGGGAGTTATTATAAC	TCCAT	ACTAC

*: Overlapping nucleotides may exist at V-D-J junction (i.e, nucleotides that could be assigned to either rearranging gene). Such nucleotides are indicated inside a parenthesis (i.e., (TACAT)) but are not included under the V, D or J gene itself.

Sub-region sequence details:

	Nucleotide sequence	Translation	Start	End
CDR3	ACCACAGATGGGGCGCTACTATGGTTCGGGGAGTTATTATAACTCCATACTACTTTGACTAC	TTDGALLWFGELL*LHTTLTT	226	287

Alignments

```
                                              <FR1-><-------CDR1-IMGT------><--------------------FR2-IMGT---------------------><--------
                                               A  S  G  F  T  F  S  N  A  W  M  S  W  V  R  Q  A  P  G  K  G  L  E  W  V  G  R  I  K  S
                            Query_1      1    GCCTCTGGATTCACTTTCAGTAACGCCTGGATGAGCTGGGTCCGCCAGGCTCCAGGGAAGGGGCTGGAGTGGGTTGGCCGTATTAAAAGC  90
V  100.0% (233/233)         IGHV3-15*01  70   ..........................................................................................  159
                                               A  S  G  F  T  F  S  N  A  W  M  S  W  V  R  Q  A  P  G  K  G  L  E  W  V  G  R  I  K  S
V  100.0% (233/233)         IGHV3-15*02  70   ..........................................................................................  159
V  99.6% (232/233)          IGHV3-15*04  70   ....................................................................................G.....  159

                                              --CDR2-IMGT---------><----------------------------------------------------FR3-IMGT--------
                                               K  T  D  G  G  T  T  D  Y  A  A  P  V  K  G  R  F  T  I  S  R  D  D  S  K  N  T  L  Y  L
                            Query_1      91   AAAACTGATGGTGGGACAACAGACTACGCTGCACCCGTGAAAGGCAGATTCACCATCTCAAGAGATGATTCAAAAAACACGCTGTATCTG  180
V  100.0% (233/233)         IGHV3-15*01  160  ..........................................................................................  249
                                               K  T  D  G  G  T  T  D  Y  A  A  P  V  K  G  R  F  T  I  S  R  D  D  S  K  N  T  L  Y  L
V  100.0% (233/233)         IGHV3-15*02  160  ..........................................................................................  249
V  99.6% (232/233)          IGHV3-15*04  160  ..........................................................................................  249

                                              --------------------------------------------><----------------------------CDR3-IMGT------
                                               Q  M  N  S  L  K  T  E  D  T  A  V  Y  Y  C  T  T  D  G  A  L  L  W  F  G  E  L  L  *  L
                            Query_1      181  CAAATGAACAGCCTGAAAACCGAGGACACAGCCGTGTATTACTGTACCACAGATGGGGCGCTACTATGGTTCGGGGAGTTATTATAACTC  270
V  100.0% (233/233)         IGHV3-15*01  250  .....................................................-------------------------------------  302
                                               Q  M  N  S  L  K  T  E  D  T  A  V  Y  Y  C  T  T
V  100.0% (233/233)         IGHV3-15*02  250  .....................................................-------------------------------------  302
V  99.6% (232/233)          IGHV3-15*04  250  .....................................................-------------------------------------  302
D  100.0% (27/27)           IGHD3-10*01  5    -------------------------------------------------------------...........................--  31
D  100.0% (20/20)           IGHD3-10*02  11   --------------------------------------------------------------------....................--  30
D  100.0% (11/11)           IGHD3-9*01   21   -----------------------------------------------------------------------------...........--  31

                                              ---------------->
                                               H  T  T  L  T  T  G  A  R  E  P
                            Query_1      271  CATACTACTTTGACTACTGGGGCCAGGGAACCCT  304
J  100.0% (31/31)           IGHJ4*02     1    ---...............................  31
J  96.8% (30/31)            IGHJ4*01     1    ---......................A........  31
J  93.3% (28/30)            IGHJ4*03     2    ----.....................A..G.....  31
```

Supplementary figure 8: Non-functional rearrangement of leukemia cells. NCBI- BLAST query output of the leukemia IGH sequence. Arrow pointing to the stop codon.

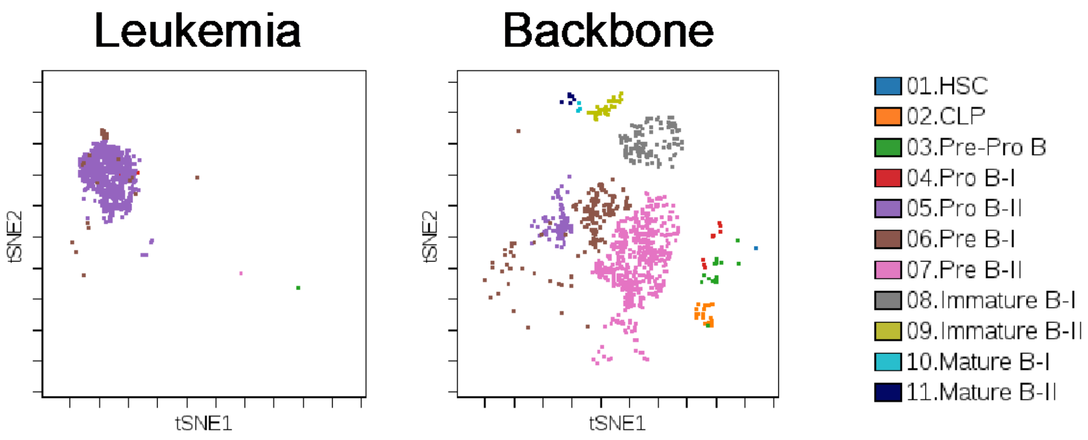

Supplementary figure 9: B-cell differentiation analysis by mass cytometry. tSNE maps of leukemic cells and engrafted cells from backbone transduced matched CB. B-cell developmental stage was analyzed by mass cytometer using developmental classifier.

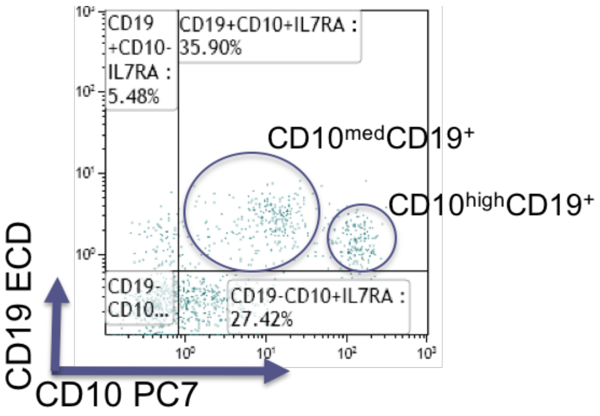

Sample	Est Total Nucleated Cells	Fraction Nucleated	nucleotide	Frequency Normalized
CD10_med CD19+_50K	19241.73	0.000185638	TGAACAGCCTGAAAACCGAGGACACAGCCGTGTATTACTGTACCACAGATGGGGCGCTACTATGGTTCGGGGAGTTATTATAA CTCCATACTACTTTGACTACTGGGGCCAGGGAACC	0.020757995
CD10highCD19low_ 4539	957.89	0.000734322	TGATTCAAAAAACACGCTGTATCTGCAAATGAACAGCCTGAAAACCGAGGACACAGCCGTGTATTACTGTACCACAGATGGGG CGCTACTATGGTTCGGGGAGTTATTATAACTCCATACTACTTTGACTACTGGGGCCAGGGAACC	0.094854162

Supplementary figure 10: Frequency of pre-leukemic clone in sorted populations from primary mouse. Viably frozen cells of the primary mouse from which the leukemic clone was developed were thawed and analyzed. Top: flow cytometry plot with marked sorted populations. Bottom table summarizing the reported frequencies of leukemic rearrangement after VH-region sequencing of genomic DNA from the sorted populations.

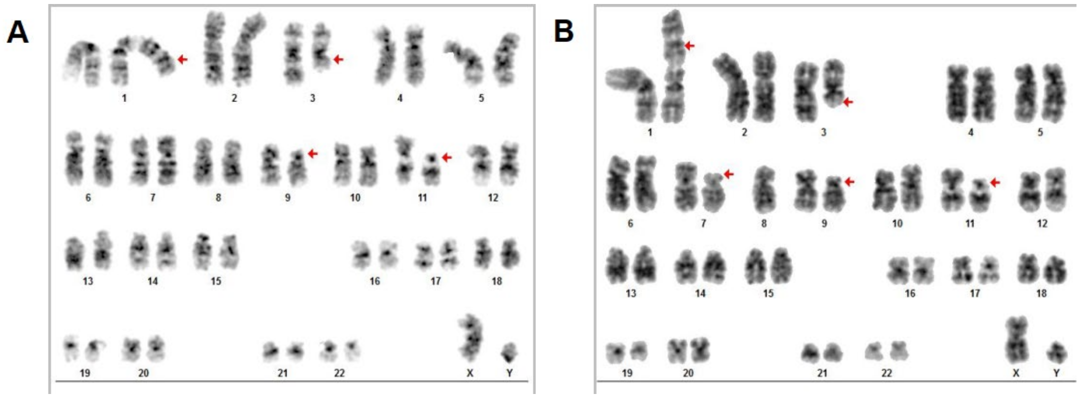

Supplementary figure 11: Karyotype analysis of leukemic cells. Leukemic cells (15 metaphases) from spleen and BM of tertiary transplanted mice were analyzed by G-banding karyotype analysis. Major clone (A) and a sub-clone (B) karyotypes with significant chromosomal aberrations (as pointed by red arrows) are:
46,XY,add(1)(p32),del(3)(q24),del(9)(p13),del(11)(p11.2)[9]/46,idem,del(7)(p13)[6].
.

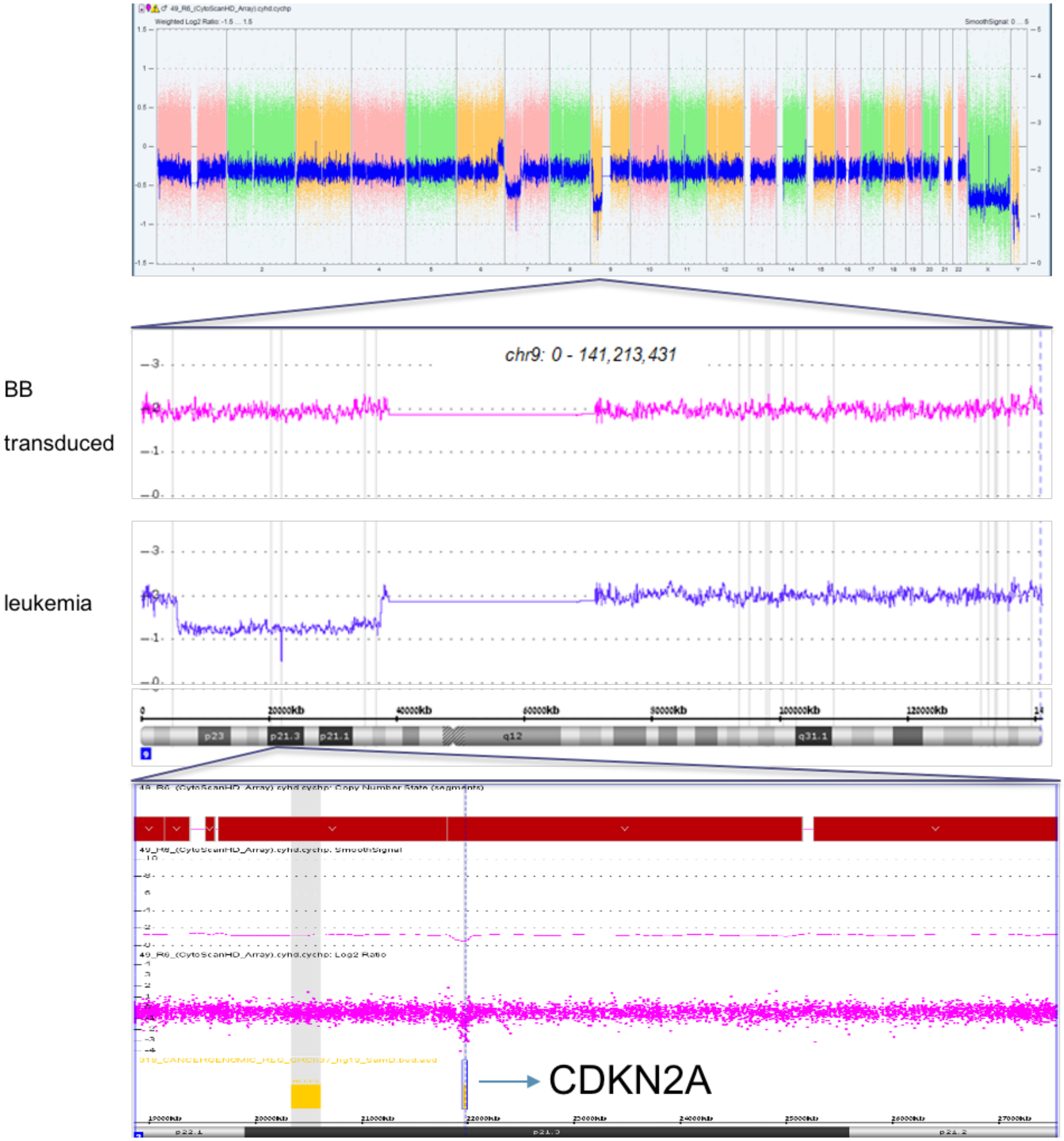

Supplementary figure 12: Bi-allelic CDKN2A deletion on chromosome 9P in Leukemic cells. Leukemic cells and backbone transduced matched cord blood were subjected to High Density Cytoscan genomic SNP array analysis. Upper image depicts whole chromosome array. Middle image analysis of chromosome 9 revealing pronounced deletion encompassing most of 9p arm in the leukemia but not in germline (BB (backbone)-transduced cord blood). Bottom image focuses on CDKN2A region with additional point deletion in CDKN2A gene.

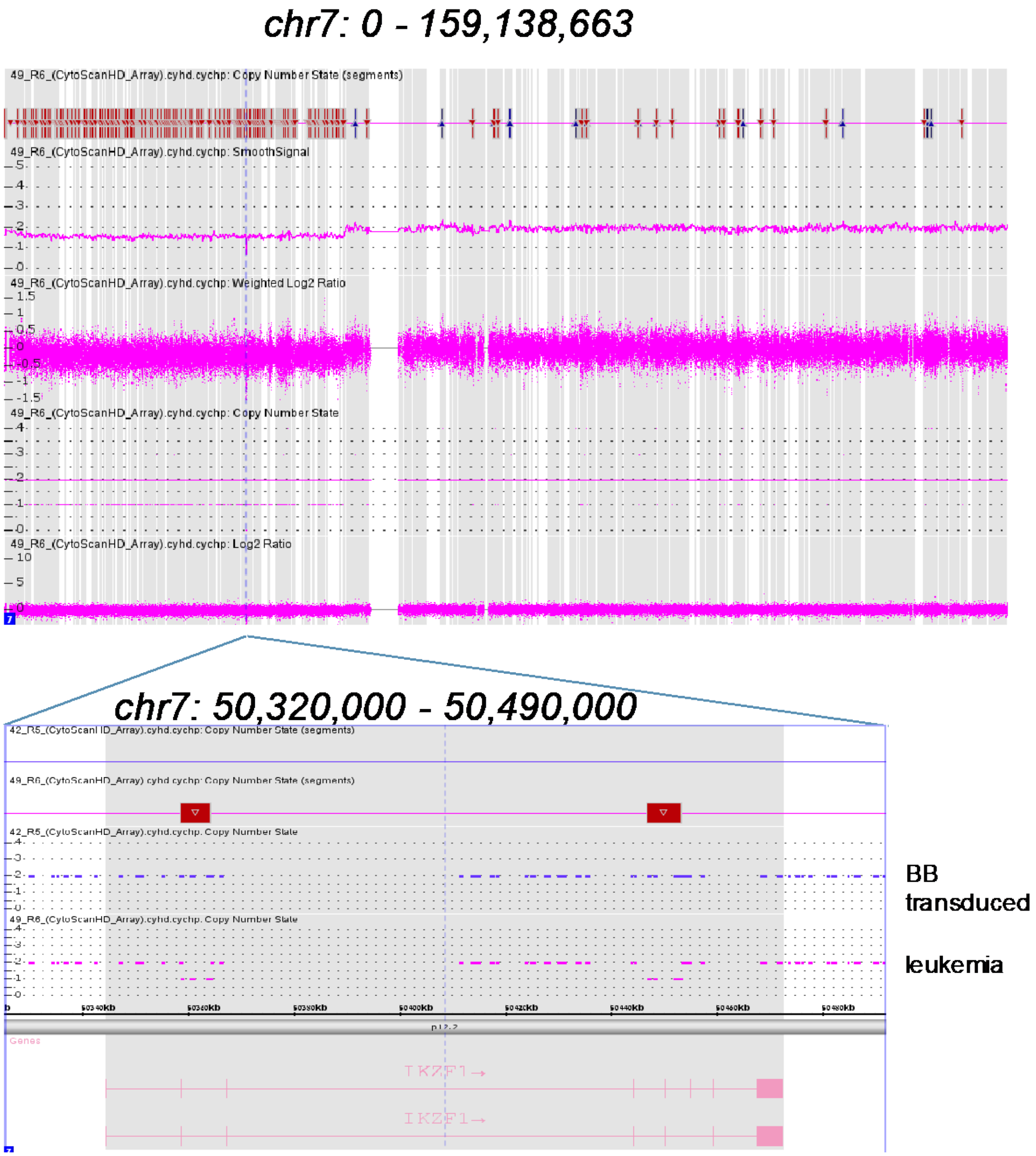

Supplementary figure 13: IKZF1 internal deletions in leukemic cells: Leukemic cells and corresponding backbone transduced and transplanted cord blood were subjected to High Density Cytoscan genomic SNP array analysis. Image depicts deletions in chromosome 7 encompassing IKZF1 region. Upper: High Density Cytoscan genomic SNP array analysis depicting chromosome 7 in whole revealing deletions around/surrounding p12.1 – p22.3. Lower: Analysis focusing on IKZF1 Genomic region depicting pronounced deletion around exons 2,5 in leukemia but not in Germline (BB (backbone)-transduced cord blood).

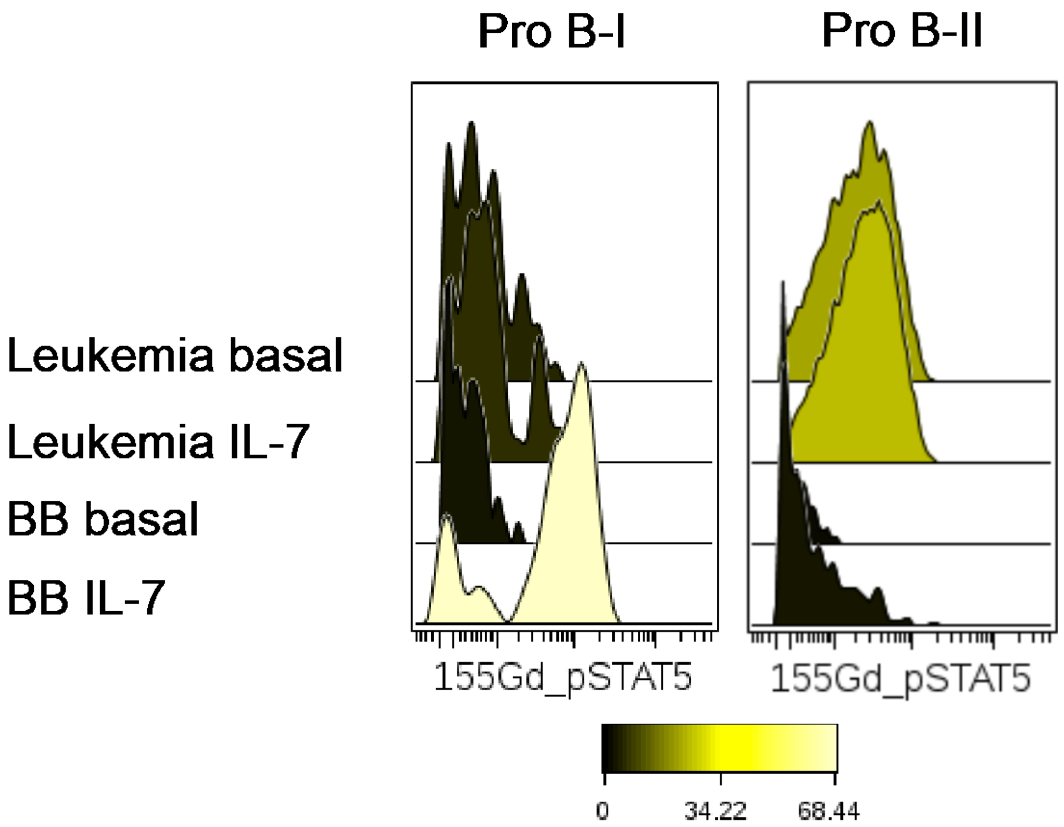

Supplementary figure 14: Cytokine independent activation of JAK-STAT signaling in leukemic cells. Histograms representing mass cytometer analysis of phosphor-STAT 5 (pSTAT5) with and without IL7 activation (100ng/ml) of ProB-I and ProB-II Leukemic cells and engrafted backbone (BB) transduced cells from matched cord blood batch. Scale bar indicates raw cytof reads.

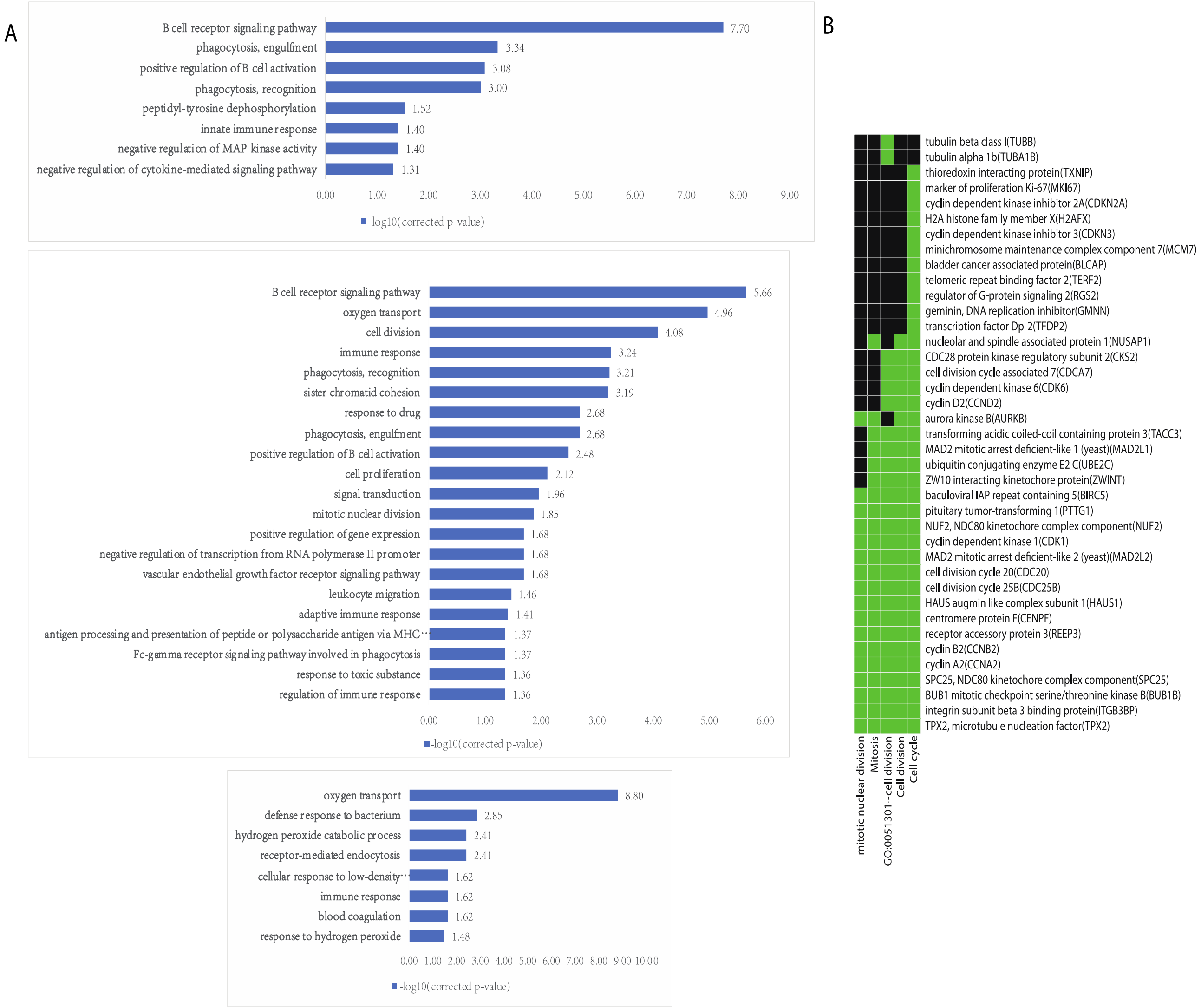

Supplementary figure 15: Functional analysis of differentially expressed genes from leukemic and pre-leukemic mice. A) Analysis of differentially expressed genes from leukemia vs. BB (top), CD10highCD19^{+} vs. BB (middle) and CD10^{+}CD19^{+} (without CD10high) vs. BB (bottom) groups. Bar graph depicting -log10 (Benjamini corrected p-values) of significantly (corrected p-value<0.05) enriched GO-Term pathways in gene ontology analysis. B) Cluster analysis of differentially expressed genes from CD10highCD19^{+} vs. BB group. 2D view image presents the top enriched cluster (enrichment score 6.52).

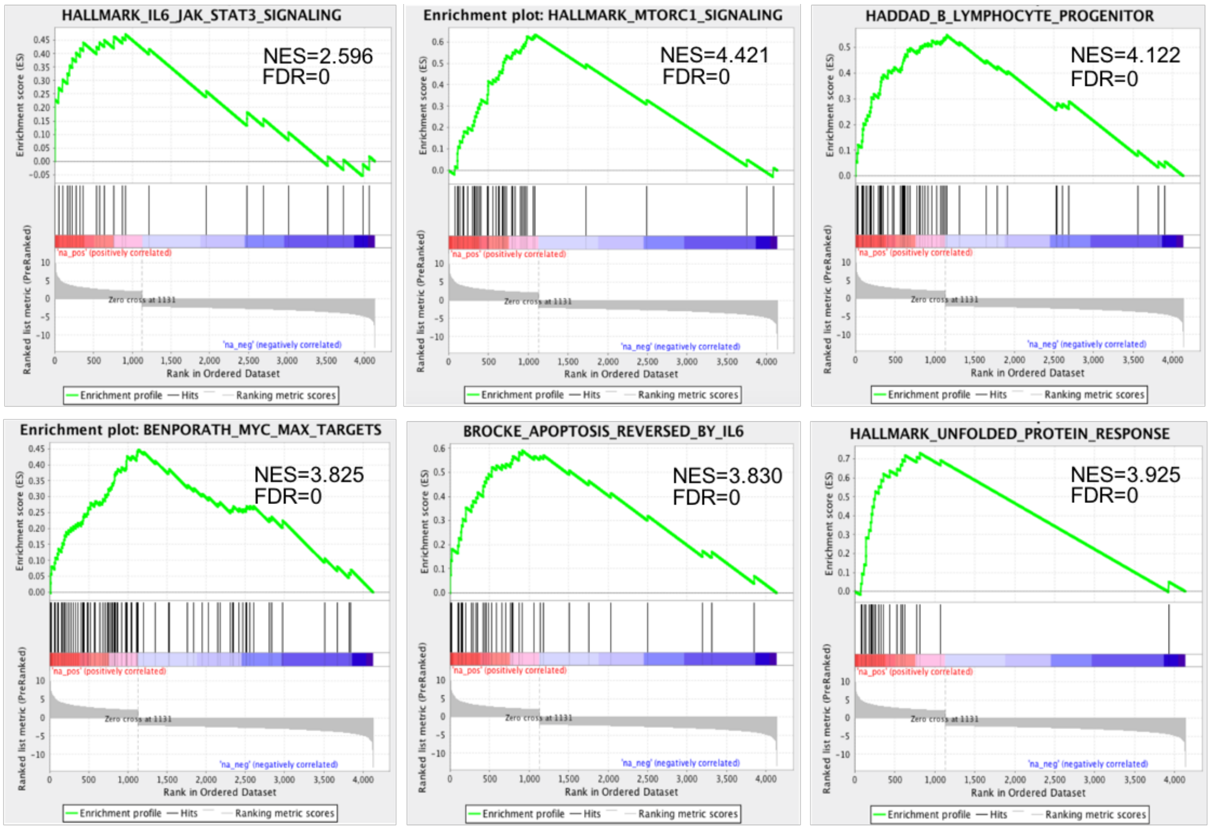

CRLF2-IL7RAins BB

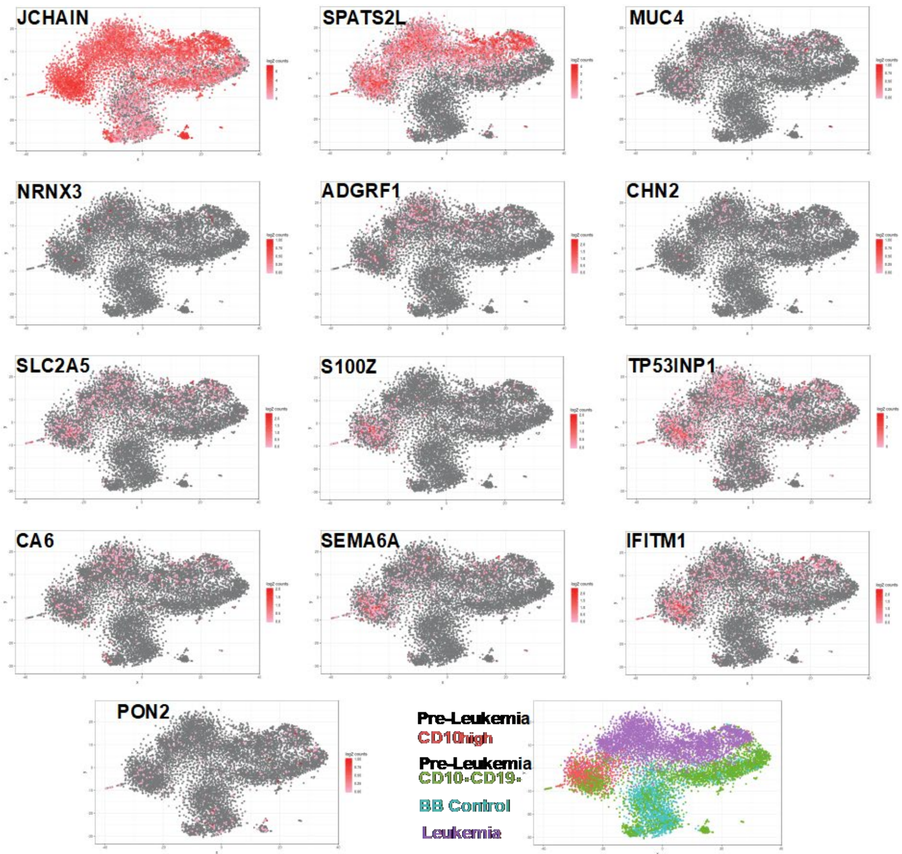

Supplementary figure 17: Philadelphia-like gene signature in Leukemia and CD10high population. Relative expression of 13 genes that were detectable by scRNAseq out of 15 ph-like diagnostic clinical panel, displayed on T-distributed somatic neighbor embedding (TSNE) map

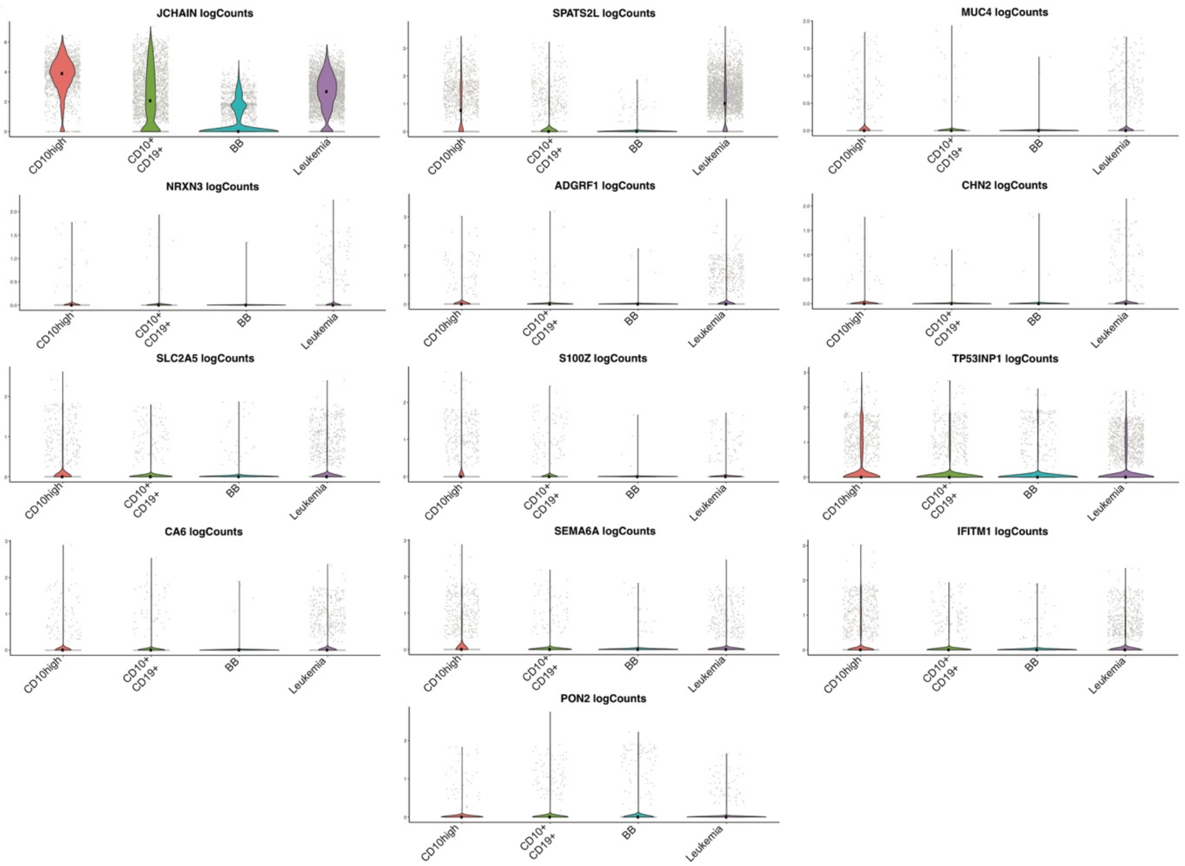

Supplementary figure 18: Philadelphia-like gene signature in Leukemia and CD10high population. Violin plot depicting the relative expression of 13 genes that were detectable by scRNAseq out of 15 ph-like diagnosis clinical panel.

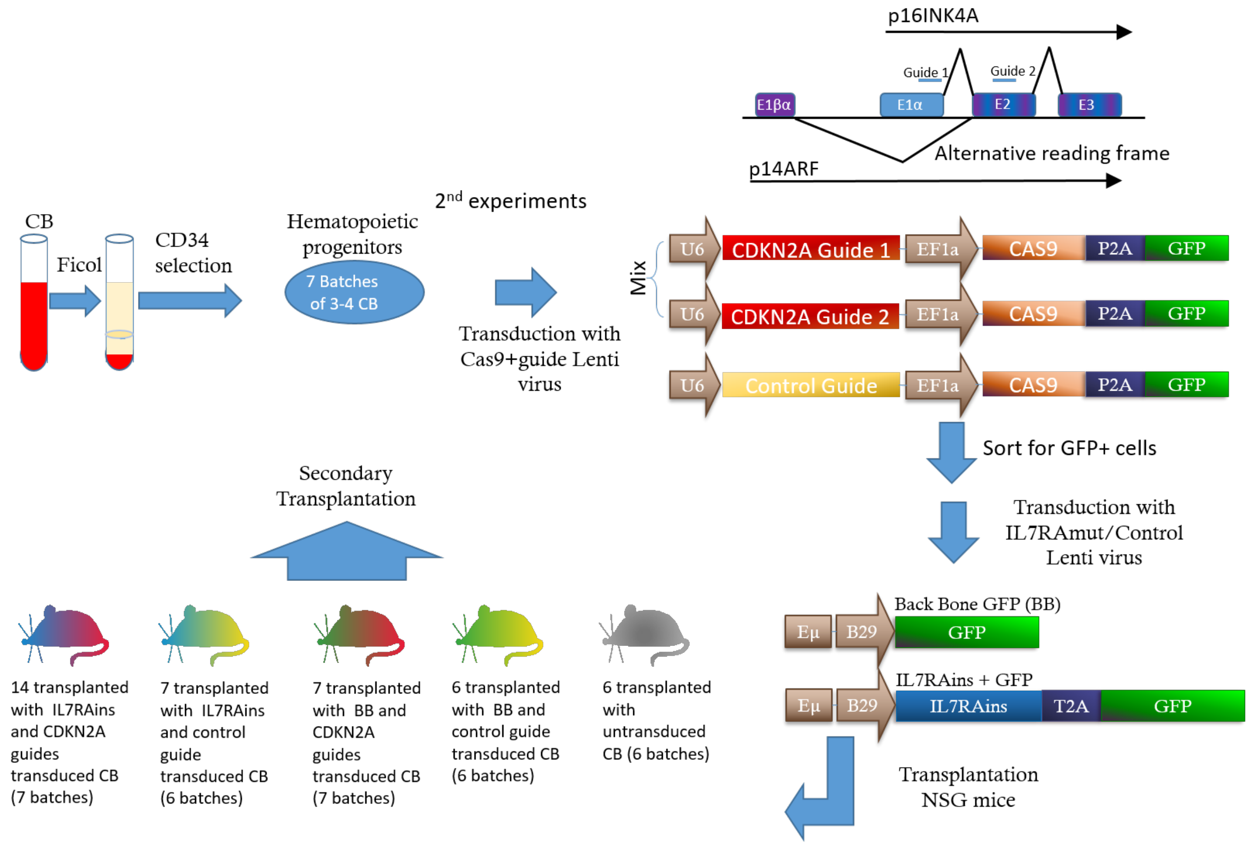

Supplementary figure 19: Experimental flow of combined IL7RA activation and CDKN2A disruption experiment: Cord Blood (CB) samples were ficolled and subjected to CD34 selection. Cells were then transduced with lentivectors directing expression of GFP and IL7RAins under B-cell minimal promoter/enhancer (pRRL Eμ B29). After secondary transduction, 3-4 transduced CB batches were combined and supplemented with negative sorted cells from the same CB batches and condition (ie GFP negative sorted cells from gCDKN2A or GFP negative sorted cells from gLucifarase were added to IL7RA/BB gCDKN2A or IL7RA/BB gLuciferase accordingly) for 50K-150K total (20-30K transduced) cells per mouse. Comparable numbers of sorted transduced cells were used per mouse from the same CB batch. Cells were transplanted in Busulfan treated (25mg/kg) 5-8 weeks old female NOD/LtSz-scid IL2Rγnull (NSG) mice. 18-26 weeks after transplantation or when showed signs of illness mice were sacrificed and cells from BM were re-transplanted into naïve Busulfan treated NSG mice.

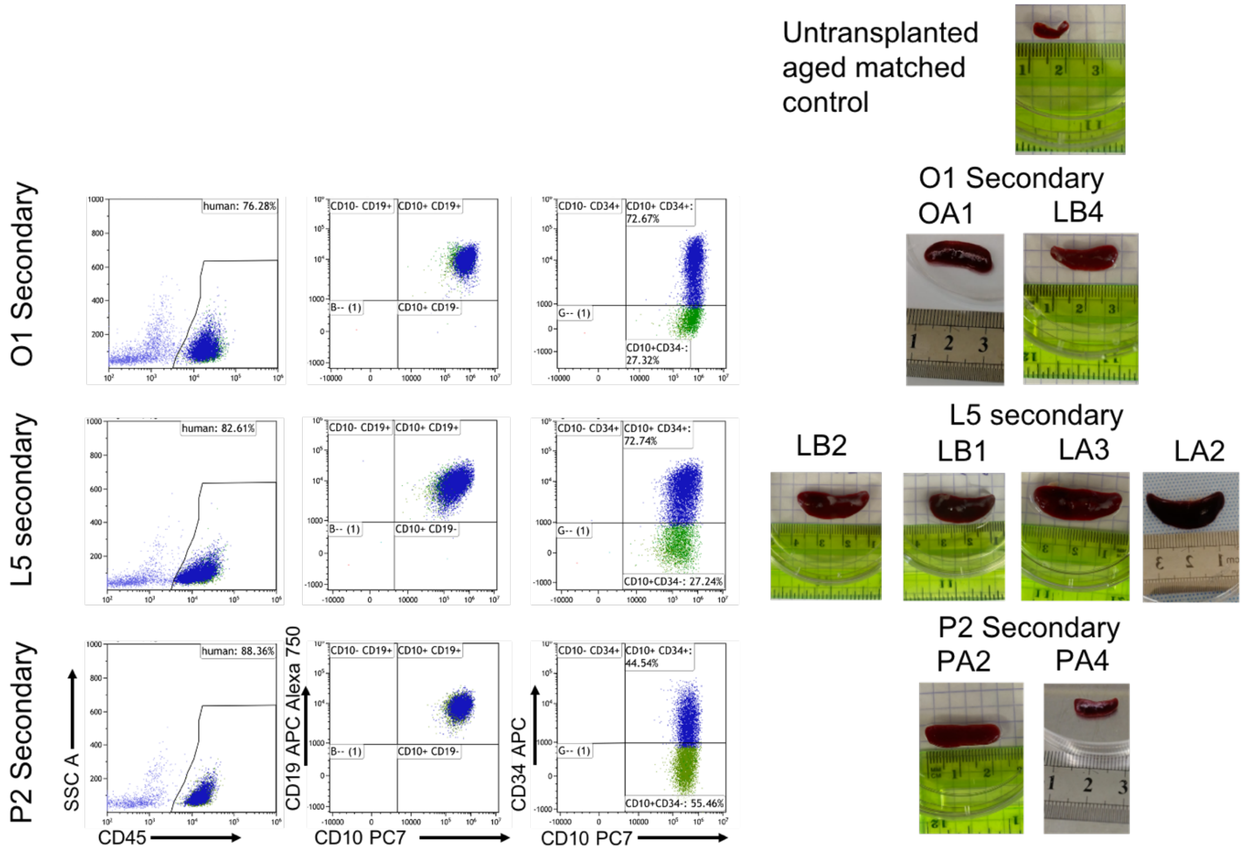

Supplementary figure 20: Human engraftment in leukemic mice. Left: Flow cytometry charts of BM from secondary mice that were transplanted with leukemic cells. Right: Pictures depicting spleen size at sacrifice of untransplanted mouse (Upper) and secondary leukemia transplanted mice.

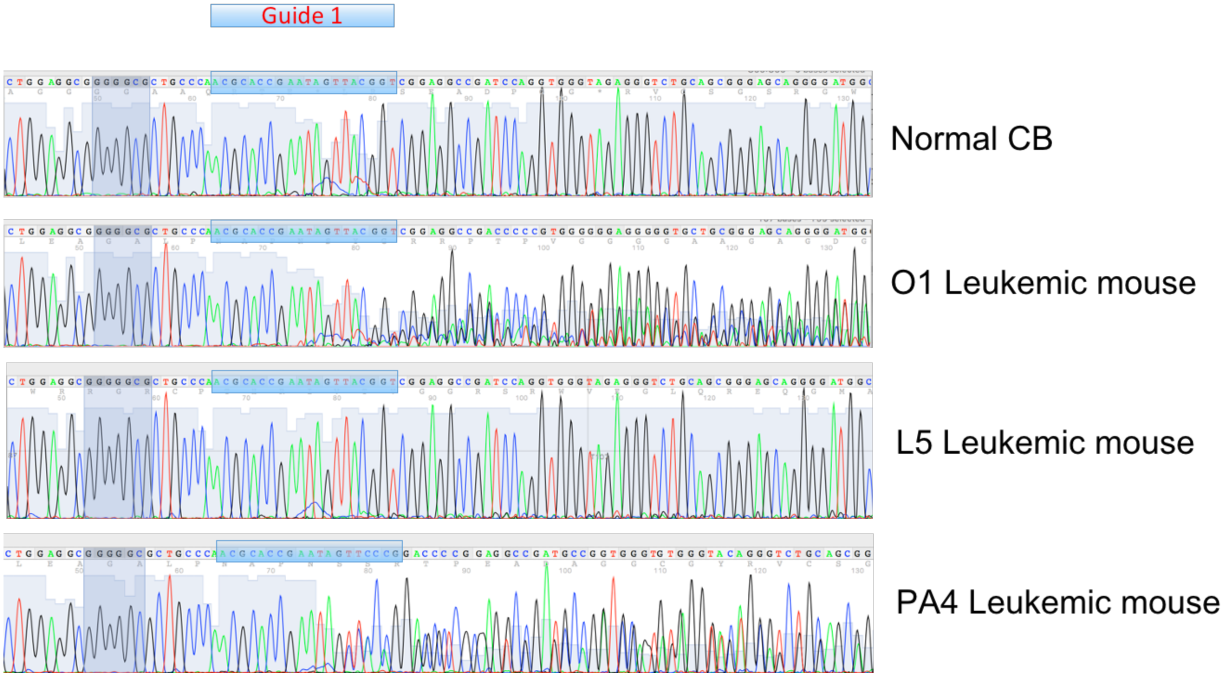

Supplementary figure 21: Editing of CDKN2A locus from Leukemic mice cells.
Sanger sequencing chromatograms of gDNA surrounding guide 1 from CD45+ cells of leukemic mice.

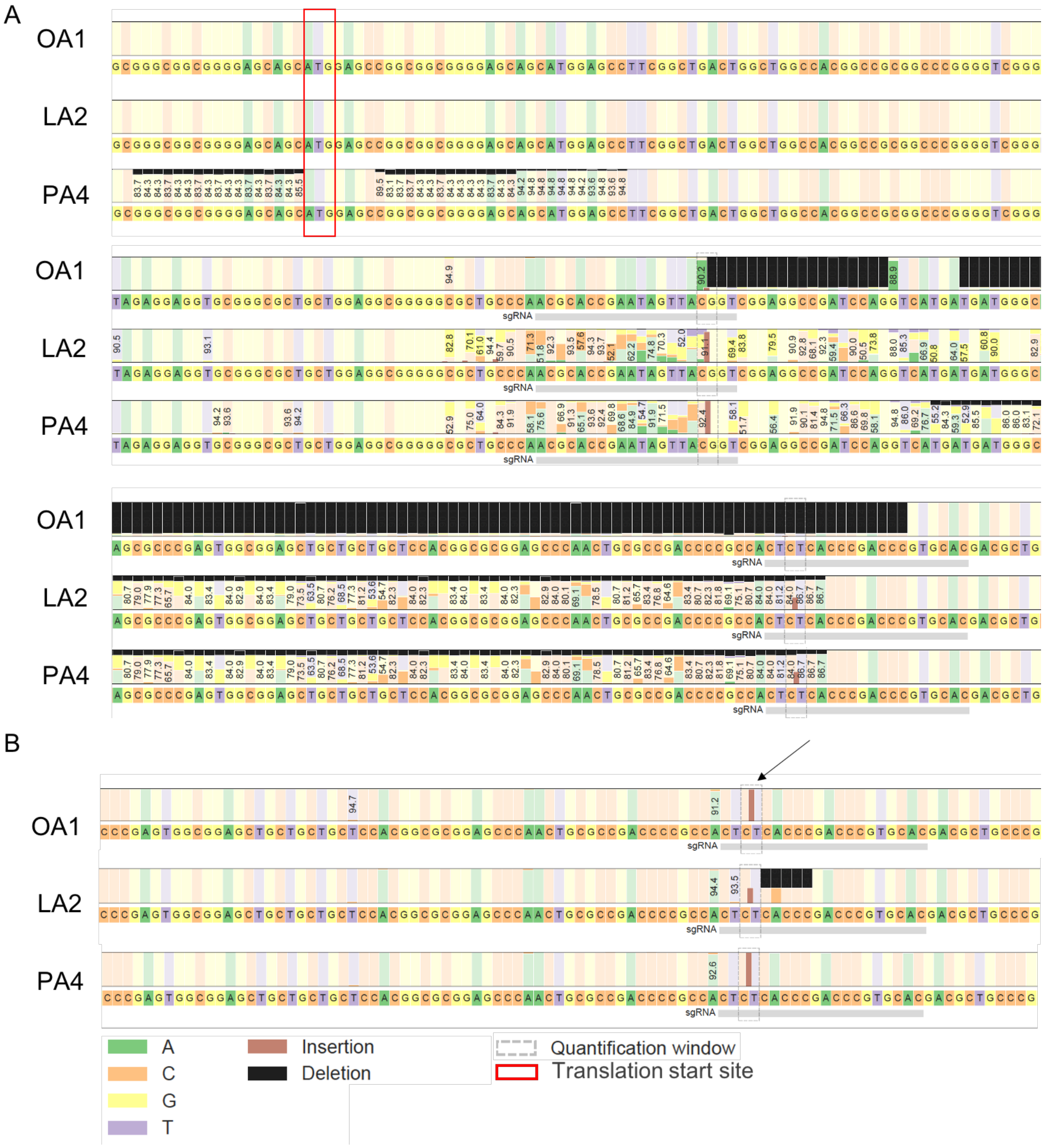

Supplementary figure 22: Sequencing of the CDKN2A gene product cDNA in leukemic cells. NGS sequencing of CDKN2A cDNA in Leukemia cells (10K depth) around guides. (A) p16INK4A (B) p14ARF. Red box indicated translation start site. Dotted gray box indicates quantification window. Black arrow indicates frame shift insertion.

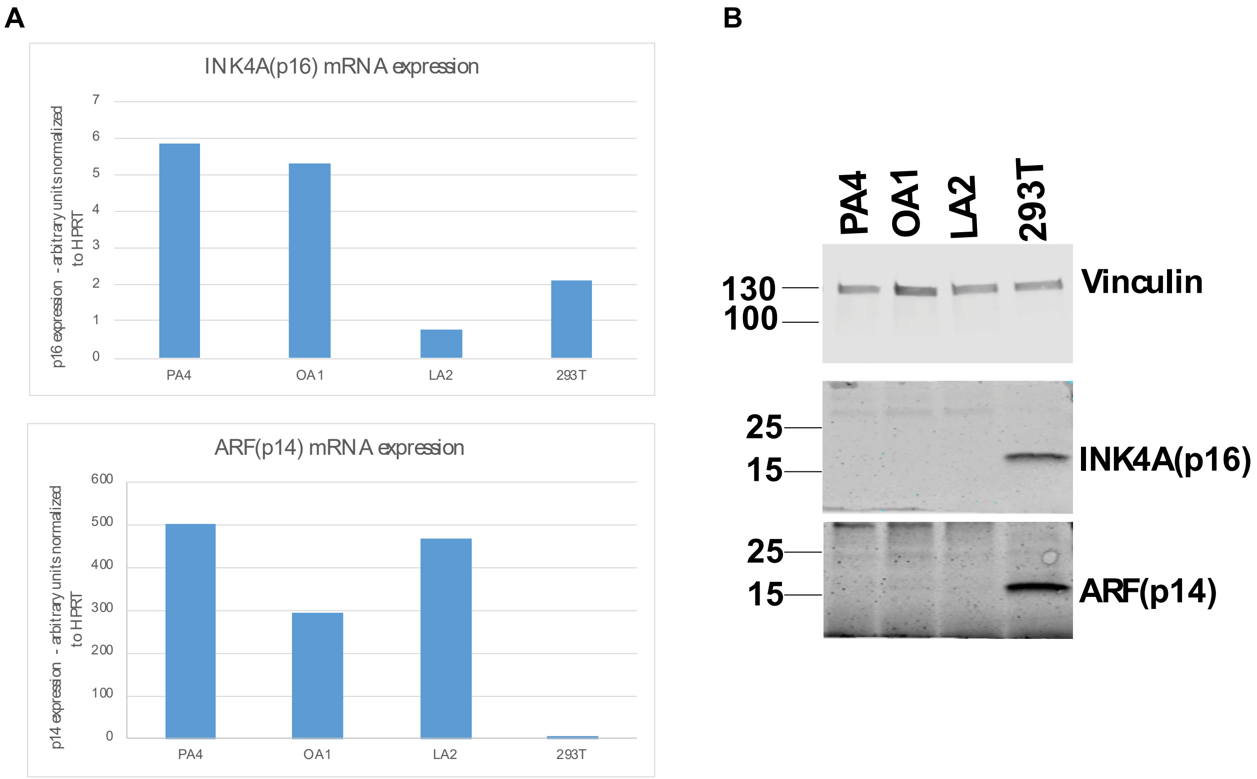

Supplementary figure 23: The CDKN2A genes INK4A p16 and ARF p14 are transcribed but no protein expression is detected. A) QPCR for p16 and p14 expression in $CD45^+$ cells from leukemic mice and 293T cells. cDNA was made from similar RNA quantities and expression was further normalized to HPRT expression. B) Western blot analysis of lysates of $CD45^+$ cells from leukemic mice and 293T cells blotted with anti p14ARF antibody and anti p16INK4A antibody. Anti-Vinculin was used as loading control. The analyses were done once. Source Data is provided in a Source Data file.

LA2

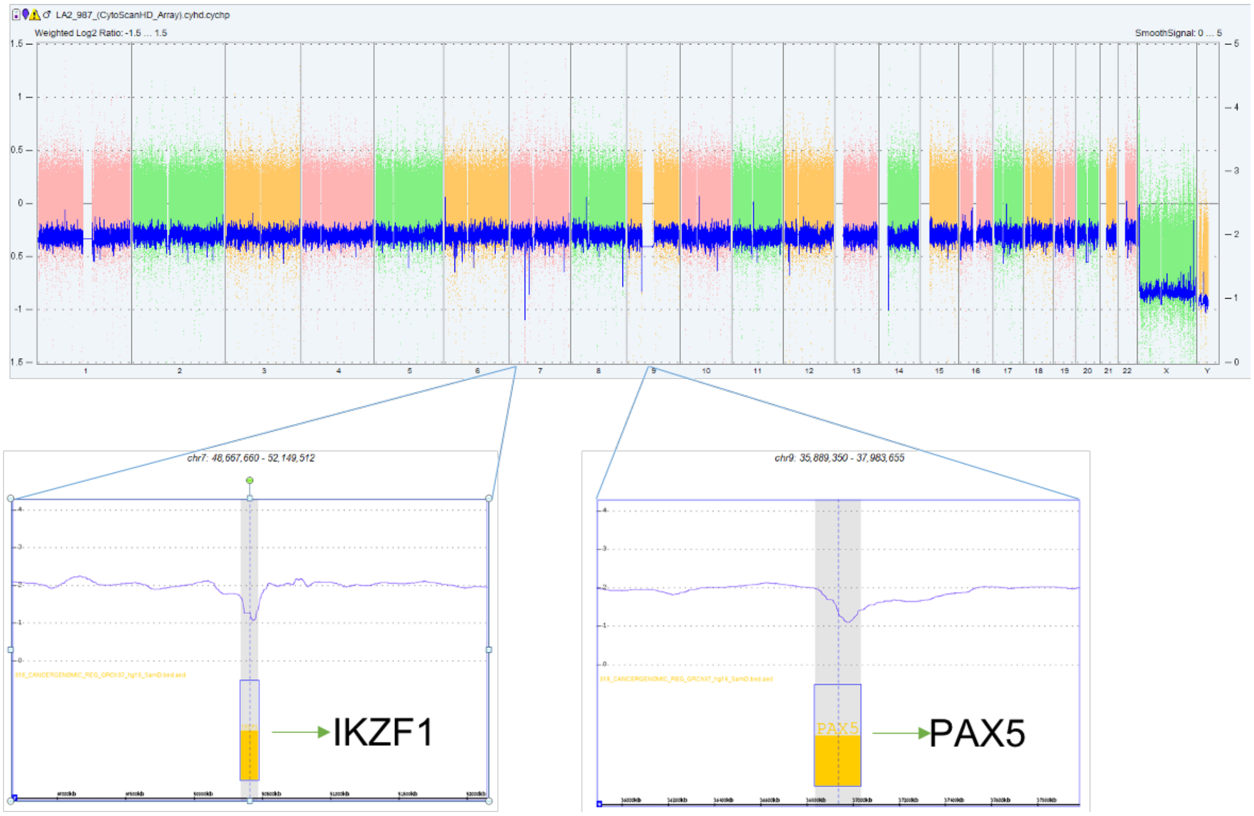

Supplementary figure 24: HD SNP array analysis of leukemias. Secondary Leukemia for L5 O1 and PA4 leukemias were analyzed by SNP array. Mono-allelic deletions of IKZF1PAX5 in L5 secondary leukemia (LA2) are depicted. SNP array of O1 and PA4 showed no abnormalities.

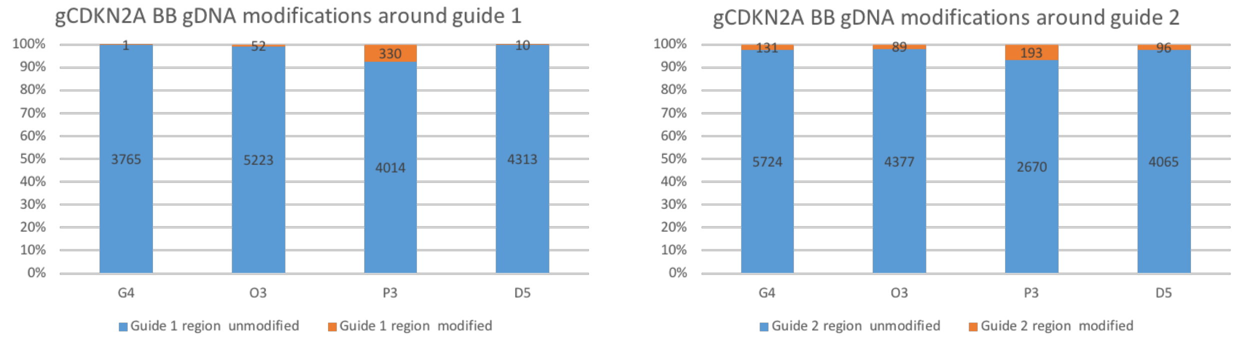

Supplementary figure 25: Editing of CDKN2A locus of transduced cells is detected in gCDKN2A-BB transplanted mice. gDNA of GFP+ sorted cells form four gCDKN2A-BB mice was subjected to NGS after PCR amplification of regions surrounding guide 1 and guide 2. Bar graphs depicting fraction of sequences of total reads. Orange: modified sequences, blue: non-modified sequences. Numbers indicate number of reads. Source Data is provided in a Source Data file.
