# Supplementary material for: An instructive role for Interleukin-7 receptor α in the development of human B-cell precursor leukemia
Source: Nat Commun. 2022 Feb 3;13:659. doi: 10.1038/s41467-022-28218-7 (PMC8814001; doi:10.1038/s41467-022-28218-7)
Supplement: Supplementary file 3 — Description of Additional Supplementary Files [file 41467_2022_28218_MOESM3_ESM.pdf]

Description of Additional Supplementary Files

Supplementary Data 1: Top differentially over expressed genes in CRLF2-IL7RAins vs backbone transduced engrafted cells. Genes are ranked according to the significance of the change (t). Fc – average of mean Log_2Fold change of paired CRLF2-IL7RAins over Backbone transduced engrafted cells from same cord blood batch.

Supplementary Data 2: Top leading genes in GSEA. Ranked list of differentially expressed genes from CRLF2-IL7RAins vs Backbone engrafted cells were analyzed for gene set enrichment (GSEA algorithm, Broad institute). Top genes leading to enrichment plot are listed.

Supplementary Data 3: Ph geneset list of differentially expressed genes from two groups of BCP-ALL: Philadelphia and Ph-like BCP-ALL versus combined groups of BCP-ALL leukemias (Patient database St. Jude's group-GSE26281) that was formed using GEO tool.

Supplementary Data 4: Top leading genes in Ph-like signature by GSEA. A gene-list of Ph-like signature was generated based on publicly available data base (St. Jude's group (GSE26281)), using the free GEO website tool- GEO2R. The list was used for gene set enrichment analysis (GSEA- broad institute) of pre-leukemia, leukemia and CRLF2-IL7RAins, compared with backbone. Top leading genes are listed.
