# Supplementary material for: An instructive role for Interleukin-7 receptor α in the development of human B-cell precursor leukemia
Source: Nat Commun. 2022 Feb 3;13:659. doi: 10.1038/s41467-022-28218-7 (PMC8814001; doi:10.1038/s41467-022-28218-7)
Supplement: Supplementary file 8 — Reporting summary [file 41467_2022_28218_MOESM8_ESM.pdf]

nature portfolio

Corresponding author(s): Shai Izraeli
Last updated by author(s): 2021/12/22

Reporting Summary

Nature Portfolio wishes to improve the reproducibility of the work that we publish. This form provides structure for consistency and transparency in reporting. For further information on Nature Portfolio policies, see our Editorial Policies and the Editorial Policy Checklist.

Statistics

For all statistical analyses, confirm that the following items are present in the figure legend, table legend, main text, or Methods section.

n/a	Confirmed	
☐	☒	The exact sample size (*n*) for each experimental group/condition, given as a discrete number and unit of measurement
☐	☒	A statement on whether measurements were taken from distinct samples or whether the same sample was measured repeatedly
☐	☒	The statistical test(s) used AND whether they are one- or two-sided<br>*Only common tests should be described solely by name; describe more complex techniques in the Methods section.*
☐	☒	A description of all covariates tested
☐	☒	A description of any assumptions or corrections, such as tests of normality and adjustment for multiple comparisons
☐	☒	A full description of the statistical parameters including central tendency (e.g. means) or other basic estimates (e.g. regression coefficient) AND variation (e.g. standard deviation) or associated estimates of uncertainty (e.g. confidence intervals)
☐	☒	For null hypothesis testing, the test statistic (e.g. *F*, *t*, *r*) with confidence intervals, effect sizes, degrees of freedom and *P* value noted<br>*Give P values as exact values whenever suitable.*
☒	☐	For Bayesian analysis, information on the choice of priors and Markov chain Monte Carlo settings
☒	☐	For hierarchical and complex designs, identification of the appropriate level for tests and full reporting of outcomes
☒	☐	Estimates of effect sizes (e.g. Cohen's *d*, Pearson's *r*), indicating how they were calculated

Our web collection on statistics for biologists contains articles on many of the points above.

Software and code

Policy information about availability of computer code

Data collection	flow cytometry and sorting: Gallios software, Cytexpert, BD FACSChorus™ Software, BD FACSDiva (versions varied due to updates through the data collection period)<br>Mass cytometry: Helios Instrument Control Software v6.
Data analysis	ImmunoSEQ IGH deep assay<br>CRISPR guides design: Genetic Perturbation Platform (Broad Institute https://portals.broadinstitute.org/gppx/crispick/public)-for CRISPR guides design<br>Crisper analysis https://crispresso.pinellolab.partners.org, VDJ rearrangements ImmunoSEQ analyzer (Adaptive biotech.com),<br>Mass cytomerty: cytobank software 7.00.<br>Expression analysis GSEA algorithm and GEO2R (GEO).<br>ScRNAseq: cellranger v.2.1.0, scatter and edgeR packages for R.<br>SNP analysis: Chromosome Analysis Suite (ChAS) 3.1.0.15<br>Microsoft Excel (versions varied due to updates through the data analysis period and several analysis computers)<br>Graph pad (versions: prism 5,prism 7)

For manuscripts utilizing custom algorithms or software that are central to the research but not yet described in published literature, software must be made available to editors and reviewers. We strongly encourage code deposition in a community repository (e.g. GitHub). See the Nature Portfolio guidelines for submitting code & software for further information.

Data

Policy information about availability of data

All manuscripts must include a data availability statement. This statement should provide the following information, where applicable:

- Accession codes, unique identifiers, or web links for publicly available datasets
- A description of any restrictions on data availability
- For clinical datasets or third party data, please ensure that the statement adheres to our policy

The WGS data generated in this study have been deposited in the European Genome-phenome archive database under accession code EGAD00001005456 https://ega-archive.org/datasets/EGAD00001005456.
The WES data generated in this study have been deposited in the European Genome-phenome archive database under accession code EGAD00001007734 https://ega-archive.org/datasets/EGAD00001007734.
The scRNAseq data generated in this study have been deposited in the Gene Expression Omnibus under accession code GSE151126, https://www.ncbi.nlm.nih.gov/geo/query/acc.cgi?acc=GSE151126.
The mass cytometry data generated in this study have been deposited in the Flow Repository under Repository ID FR-FCM-Z4XM (https://flowrepository.org/id/FR-FCM-Z4XM).
The bulk RNAseq data generated in this study have been deposited in the Gene Expression Omnibus under accession code GSE190070, https://www.ncbi.nlm.nih.gov/geo/query/acc.cgi?acc=GSE190070
The NGS data generated in this study have been deposited in the Gene Expression Omnibus under accession code GSE190070, https://www.ncbi.nlm.nih.gov/geo/query/acc.cgi?acc=GSE190070
The Single Nucleotide Polymorphism (SNP) array data generated in this study have been deposited in The European Bioinformatics Institute (EMBL-EBI) ArrayExpress –under ID E-MTAB-11258 https://www.ebi.ac.uk/arrayexpress/experiments/E-MTAB-11258/
Source data are provided with this paper.

Field-specific reporting

Please select the one below that is the best fit for your research. If you are not sure, read the appropriate sections before making your selection.

☒ Life sciences ☐ Behavioural & social sciences ☐ Ecological, evolutionary & environmental sciences

For a reference copy of the document with all sections, see nature.com/documents/nr-reporting-summary-flat.pdf

Life sciences study design

All studies must disclose on these points even when the disclosure is negative.

Sample size	For quantity assays mice experiments group numbers were planned using G*power 3 (free open software https://www.psychologie.hhu.de/arbeitsgruppen/allgemeine-psychologie-und-arbeitspsychologie/gpower.html). Assuming medium effect (f=0.5), a=0.05, power 0.95, six groups. The calculated total sample size was 85. (~15 mice/ group). Anticipating mice death, engraftment variability, transduction variability and cord blood sample variability mice numbers per group was increased up to 25 mice/group, depending on virus and cord blood sample availability.
Data exclusions	In immunophenotyping analyses, samples with less than 2% hCD45 and or less than 3% distinct transduced CD19+ population were excluded duo to inaccuracy of the measurement. Additionally, samples with GFP that was too bright to analyze (due to overspill that could not be corrected with compensation) were omitted.<br>In single cell RNAseq analysis Low quality cells were filtered out by discarding cells that failed either one of these criteria: 1. More than 10% of the cells' detected genes were mitochondrial. 2. Cells had less than 2 median absolute deviations (MADs) of detected genes (less than 455 genes). 3. Cells had less than 2 MADs log10 total counts (less than 728 counts).
Replication	The Experimental development of leukemia described in this paper repeated in four out of the 22 cord blood batches that were used in this manuscript. This incidence rate is within acceptable range for development of leukemia in human system. Different analyses on the leukemic samples such as sequencing and differentiation assessment were done once.
Randomization	Each batch of cord blood was split equally for several experimental groups. Control group was included in each batch.<br>Mice were marked and transplanted randomly by technician who was blind to experimental parameters.
Blinding	All transplantations were done by a technician who was blind to group allocation.<br>Flow cytometry data collection and analysis was done by the first author and thus blinding was not possible.<br>Receptor editing, SNP analysis and single cell analyses were done by investigators blinded to the group allocation

Reporting for specific materials, systems and methods

We require information from authors about some types of materials, experimental systems and methods used in many studies. Here, indicate whether each material, system or method listed is relevant to your study. If you are not sure if a list item applies to your research, read the appropriate section before selecting a response.

Materials & experimental systems

n/a	Involved in the study
☐	☒ Antibodies
☐	☒ Eukaryotic cell lines
☒	☐ Palaeontology and archaeology
☐	☒ Animals and other organisms
☐	☒ Human research participants
☒	☐ Clinical data
☒	☐ Dual use research of concern

Methods

n/a	Involved in the study
☒	☐ ChIP-seq
☐	☒ Flow cytometry
☒	☐ MRI-based neuroimaging

Antibodies

Antibodies used

flow cytometry antibodies:
antigen; Clone; Manufacturer; fluorophore; Dilution; Cat number;
CD45; 5B1; Miltenyi; Vio Green; 1:50; 130-096-906;
CD45;HI30; Biolegend; Pacific Blue; 1:50; 304029;
CD45;HI30; Biotest; APC 1:50; 17-0459-42;
CD127(IL7RA); R34.34; Beckman Coulter; APC-Alexa 700; 2:50; A71116;
CD127(IL7RA); eBioRDR5; eBioscience; Super Bright 780; 2:50; 78-1278-42;
CD127(IL7RA); A019D5; Biolegend; BV421; 2:50; 351310;
CD19; J3-119; Beckman Coulter; ECD; 1:50; A07770;
CD19; J3-119; Beckman Coulter; APC-Alexa750; 1:50; A78838;
IgM; CH2; exbio; APC; 1:100; 1A-320-C100;
CD10; ALB1; Beckman Coulter; PC7; 1:50; A465527;
CD34; BIRMA-K3; DAKO/Agilent; RPE; 1:100; C723850-2;
CD34; 8G12/HPCA2; BD Pharmingen; APC; 1:100; 345804;
CD16/32; 93; Biolegend; none; 1:50; 101326;
TSLPR; ID3; Biolegend; PE; 1.5:50;322906;
TSLPR; IB4; Biolegend; PE; 1.5:50; 322806;

mass cytometry:
Protein; Clone; Manufacturer; Metal Isotope; Staining; Diluition;
(in 100 µL)
4EBP1(pT36/T46); 236B4; Cell Signaling Technology; Nd144; Intracellular; 1 µL;
Akt (pS473); D9E; Cell Signaling Technology ;Tb159; Intracellular; 1 µL
BTK (pY551/511); 24A/BTK; BD Biosciences; Yb174; Intracellular; 2 µL
cCaspase3; C92-605; BD Biosciences; Ho165; Intracellular; 1 µL
CD10; HI10a; Biolegend; Gd156; Surface; 1 µL
CD127; A019D5; Biolegend; Dy162; Surface; 1.5 µL
CD16; 3G8; Fluidigm; Bi209; Surface; 2 µL
CD179a; HSL96; Biolegend; Sm149; Intracellular; 0.3 µL
CD179b; HSL11; Biolegend; Gd158; Intracellular; 0.5 µL
CD19; H1B19; Biolegend; Nd142; Surface; 0.5 µL
CD20; 2H7; Biolegend; Sm147; Surface; 0.5 µL
CD22; HIB22; Biolegend; Nd143; Surface; 1 µL
CD235; HIR2; Biolegend; In115; Surface; 0.25 µL
CD24; ML5; Biolegend; Gd160; Surface; 1 µL
CD3; UCHT1; Biolegend; Er170; Surface; 0.25 µL
CD34; 581; Biolegend; Nd148; Surface; 0.25 µL
CD38; HIT2; Biolegend; Er168; Surface; 0.5 µL
CD43; CD43-10G7; Biolegend; Er167; Surface; 0.25 µL
CD45; human; HI30; Fluidigm; Y89; Surface; 0.25 µL
CD45; mouse; 30F11; Biolegend; In113; Surface; 0.125 µL
CD79b; CB3-1; Biolegend; Nd146; Surface; 0.5 µL
cPARP; F21-852; BD Biosciences; La139; Intracellular; 0.25 µL
Creb (pS133); 87G3; Cell Signaling Technology; Yb176; Intracellular; 5 µL
CRLF2; 1A6; eBioscience; Dy161; Surface; 1 µL
CyclinA (total); BF-683; BD Biosciences; Sm154; Intracellular; 0.25 µL
CyclinB1 (total); GNS-1; BD Biosciences; Dy164; Intracellular; 1 µL
Erk1/2 (pT202/pY204); D13-14-4E; Cell Signaling Technology; Yb173; Intracellular; 1 µL
Glucocorticoid Receptor; D8H2; Cell Signaling Technology; Eu151; Intracellular; 2 µL
GFP; SF12.4; Fluidigm; Tm169; Intracellular; 1 µL
HistoneH3 (pS28); HTA28; Biolegend; Ce140; Intracellular; 0.25 µL
IgHintracellular; polyclonal; Novus; Eu153; Intracellular; 0.5 µL
IgH surface; MHM-98; Fluidigm; Yb172; Surface; 0.25 µL
Ikaros (total); D10E5; Cell Signaling Technology; Nd145; Intracellular 2 µL
Ki67; B56; BD Biosciences; Sm152; Intracellular; 1 µL
PU.1 9G7; Cell Signaling Technology; Gd157; Intracellular 1 µL
RB (pS807/811); J112-906; BD Biosciences; Er166; Intracellular; 0.25 µL
rpS6; (pS235/pS236); N7-548; BD Biosciences; Lu175; Intracellular; 1 µL

SRC (pY418); K98-37; BD Biosciences; Pr141; Intracellular; 2 µL
STAT5 (pY694); 47; BD Biosciences; Gd155; Intracellular; 0.5 µL
Syk; (pY319/pY352); 17a; BD Biosciences; Yb171; Intracellular; 1 µL
TdT; E17-1519; BD Biosciences; Dy163; Intracellular; 1 µL

Western bloting:
vinculin (1:40,000, MAB3574 millipore Sigma), CDKN2A/p16INK4A [EPR1473] (1:500, ab108349, Abcam), CDKN2A/p14ARF [EPR17878] (1:500, ab185650, Abcam), Goat anti Rabbit IgG H&L (1:10000, IRDye® 800CW preadsorbed, ab216773, Abcam) and Goat anti-Mouse IgG H&L (1:10000, IRDye® 800CW) preadsorbed ab216772, Abcam).

Validation

All antibodies were previously published and established. most are used for diagnostic clinical purposes. No new antibodies/clones were used in this research.

Eukaryotic cell lines

Policy information about cell lines

Cell line source(s)

293T - ATCC

Authentication

Cell line was not authenticated as it was used for preparation of virus and not as research line.

Mycoplasma contamination

Cells were subjected to periodical mycoplasma tests and were confirmed negaive

Commonly misidentified lines
(See ICLAC register)

Name any commonly misidentified cell lines used in the study and provide a rationale for their use.

Animals and other organisms

Policy information about studies involving animals; ARRIVE guidelines recommended for reporting animal research

Laboratory animals

Immune deficient NOD/LtSz-scid IL2Rγnull female of 5-8 weeks were used for transplantations. Mice were bred and housed in specific pathogen-free conditions, In an Techniplast IVC cage system with temperature range 19-210C humidity range 45-65% 11.5 light 12.5 dark hours.

Wild animals

The study did not involve wild animals.

Field-collected samples

The study did not involve samples collected from the field

Ethics oversight

All animal experiments were approved by the Animal Care Committee at Sheba Medical Center (IRB 1007/15) and animal care committee at Rabin Medical Center (022_b15189 040419)Human research participants

Note that full information on the approval of the study protocol must also be provided in the manuscript.

Human research participants

Policy information about studies involving human research participants

Population characteristics

Randomized cord blood units that are otherwise discarded due to insufficient volume for public storage were used in the research. The samples are stripped of any information (ID, birth term, sex, genotype)

Recruitment

Donations of cord blood to public cord blood bank are recruited among labors in the obstetric delivery department. Informed consent is signed that cord blood specimens that are not suitable for banking will be used for research. No compensation is granted upon donation.

Ethics oversight

Institutional review board, Sheba medical center (Approval 5638-08-SMC)

Note that full information on the approval of the study protocol must also be provided in the manuscript.

Flow Cytometry

Plots

Confirm that:

☒ The axis labels state the marker and fluorochrome used (e.g. CD4-FITC).

☒ The axis scales are clearly visible. Include numbers along axes only for bottom left plot of group (a 'group' is an analysis of identical markers).

☒ All plots are contour plots with outliers or pseudocolor plots.

☒ A numerical value for number of cells or percentage (with statistics) is provided.

Methodology

Sample preparation	Hematopoietic tissues (Spleen and Bone marrow (BM)) were harvested from mice at sacrifice time and kept throughout the processing time on ice. BM cells were flashed from the hind leg bones and strained through a 70µm mesh cell strainer. Spleens were mashed on a 70µm mesh cell strainer. Spleens were subjected to red blood cell lysis (Biolegend, San Diego, CA, USA) per manufacturer's protocol. Cells that were not used for immediate analysis/sort were viably frozen in FBS+10% DMSO. Standard staining protocols were used for sort and analysis of cells. In brief, cells were washed in staining media (2% FBS in PBS) and re-suspended in of staining media containing fluorochrome-conjugated antibodies, blocking antibodies when mouse tissue was used and 7AAD for 30 min. Following staining, cells were washed and analyzed/ sorted.
Instrument	Data was collected using Gallios flow cytometer and Cytoflex flow cytometer (Beckman-Coulter, California, USA). Sorting was done using ARIA I/Aria III /Melody FACS sorters (BD Biosciences, San Jose, CA USA). Single stains and FMOs (Full minus one staining) of each fluorophore were used for cytometer setup and gating.
Software	Analysis was preformed using Kaluza/ software (Beckman-Coulter, California, USA)
Cell population abundance	For samples rich in target populations purity of the sorted population was determined by sampling of sorted cells and re-run on the sorter. Using same gates we validated that collected cell population falls within target gates. Purity of the sorter samples was consistently greater than 90%. When sorting rare populations for Gene expression analysis, validating purity by flow could not be done, however, after analysis, expression of the sorting markers was validated (for example for transduced cells, expression of the coded transgene)
Gating strategy	Single stains and FMOs (Full minus one staining) of each fluorophore were used for cytometer setup and compensations.<br>Cord blood sample depleted of CD34 cells was used to determine positive human markers.<br>Specifically: FCS/SSC parameters were set according to human cord blood sample and untransplanted mouse sample in each xenograft analysis. Using FMO for 7AAD a "live" cell gate was drawn followed by CD45 positive gate that was determined using untransplanted mouse sample. An untraduced/different transgene sample was used to determine pos/neg gates for each transgene (Untraduced transplanted sample for GFP pos/neg population, backbone GFP transduced/untransduced sample for CRLF2/IL7RA pos/neg population) . Transduced populations were then immunophenotyped by cell surface markers whose gates were set using FMO staining of each marker.

☒ Tick this box to confirm that a figure exemplifying the gating strategy is provided in the Supplementary Information.
